# Leaf phenology determines the response of poplar genotypes to O_3_ through mesophyll conductance

**DOI:** 10.1111/tpj.70091

**Published:** 2025-03-07

**Authors:** Yasutomo Hoshika, Elena Paoletti, Claudia Pisuttu, Lorenzo Cotrozzi, Matthew Haworth, Elisa Pellegrini, Cristina Nali, Rafael Vasconcelos Ribeiro, Juliana Lischka Sampaio Mayer, Barbara Baesso Moura

**Affiliations:** ^1^ Institute of Research on Terrestrial Ecosystems (IRET) National Research Council of Italy (CNR) Via Madonna del Piano Sesto Fiorentino I‐50019 Italy; ^2^ NBFC, National Biodiversity Future Center Palermo 90133 Italy; ^3^ Department of Agriculture, Food and Environment University of Pisa Via del Borghetto 80 Pisa 56124 Italy; ^4^ CIRSEC, Centre for Climate Change Impact University of Pisa Via del Borghetto 80 Pisa 56124 Italy; ^5^ Institute for the Sustainable Plant Protection (IPSP) National Research Council of Italy (CNR) Via Madonna del Piano Sesto Fiorentino I‐50019 Italy; ^6^ Department of Plant Biology, Institute of Biology State University of Campinas (UNICAMP) Campinas São Paulo Brazil

**Keywords:** mesophyll conductance, ozone, carbon assimilation, structural equation modelling, photosynthesis, *Populus*, leaf age, leaf anatomy, ABA, ozone FACE

## Abstract

Tropospheric ozone (O_3_) is a phytotoxic air pollutant that impairs photosynthesis. The mechanisms of O_3_‐induced reduction of mesophyll conductance (*g*_m_) are not clear. We investigated the interaction of O_3_ and leaf age on *g*_m_ by using structural equation modelling (SEM) for two poplar clones (I‐214 and Oxford) exposed to three O_3_ levels (ambient air, AA; 1.5 × AA; 2.0 × AA) in a free‐air controlled experiment. Clone‐specific phenological responses to elevated O_3_ were found: I‐214 showed a rapid leaf turnover and formed new productive leaves, whereas Oxford was more ‘conservative’ maintaining old or injured leaves. In the I‐214 clone with fast leaf turnover, *g*_m_ was reduced due to increasing cell wall thickness in new leaves, a possible reaction to increase its resistance against O_3_ damage. As I‐214 leaves aged, a decrease in the fraction of the mesophyll surface area unoccupied by chloroplasts was observed at 2.0 × AA prior to a reduction in photosynthesis. In the Oxford clone with slow leaf turnover, *g*_m_ was mainly affected by physiological rather than structural factors: in particular, a marked reduction of *g*_m_ caused by abscisic acid (ABA) was noticed. As photosynthesis is limited by diffusional barriers, O_3_ effects on *g*_m_ will be key for carbon sequestration modelling of O_3_ pollution and climate change.

## Significance Statement

Ozone‐induced reductions in the mesophyll conductance of poplar were caused by leaf structural acclimation in clones with fast leaf turnover, but by ABA in clones with longer leaf lifespans.

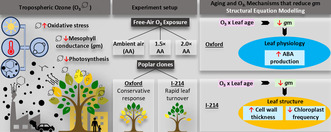

## INTRODUCTION

Ozone in the troposphere (O_3_) is a significant greenhouse gas and phytotoxic air pollutant, and its concentration has risen in the Northern Hemisphere since the 19th century (Mills et al., 2018). Although peak O_3_ concentrations have recently decreased in some regions due to reduced emissions of O_3_ precursors, the ambient O_3_ concentration is still sufficient to cause negative impacts on terrestrial plants (De Marco et al., 2022; Mills et al., 2018).

Once O_3_ enters into plants through stomata, its phytotoxicity causes damage to physiological processes, such as photosynthesis (Grulke & Heath, 2020); although the sensitivity of plants to O_3_ depends upon species and variety (Cheesman et al., 2023; Li et al., 2017; Osborne et al., 2016). One of the important factors determining plant resistance to O_3_‐induced oxidative stress is growth pattern (Hoshika, Carrari, et al., 2020; Manninen et al., 2009; Pell et al., 1995), which depends on plant phenological characteristics, in particular flushing or successive leaf growth patterns (Hoshika, Carrari, et al., 2020; Koike, 1995). Plants with successive patterns exhibit continuous shoot growth, which may permit shedding of damaged leaves and formation of new leaves to maintain carbon assimilation under elevated O_3_, while plants with a flushing pattern show a ‘conservative’ response by maintaining old leaves and tolerating O_3_ stress (Hoshika, Carrari, et al., 2020; Tjoelker & Luxmoore, 1991). Ozone damage to photosynthesis depends on leaf age. Indeed, young leaves in plants with a successive pattern may show a relatively high net photosynthetic rate under elevated O_3_ conditions, whereas old leaves have been observed to show a significant decrease in photosynthetic capacity due to premature senescence (Hoshika, Watanabe, et al., 2013; Noormets et al., 2001; Yuan et al., 2020).

Mechanisms of O_3_ effects on photosynthesis are complex. Ozone may decrease nitrogen (N) use efficiency in association with reduced activity of ribulose‐1,5‐bisphosphate carboxylase/oxygenase (RubisCO) (Bagard et al., 2015). Also, O_3_ may affect stomatal regulation and leaf water status, which may increase CO_2_ diffusion resistance inside the leaves (Hoshika, Fares, et al., 2020; Kitao et al., 2009). The movement of CO_2_ across the mesophyll layer has become an increasingly important characteristic in elucidating plant responses to abiotic stress (e.g. Brunetti et al., 2019). Several recent studies have dealt with the importance of mesophyll conductance (*g*_m_) to O_3_‐induced reductions in photosynthesis in hybrid poplar clones (Joffe et al., 2022; Xu et al., 2019), Japanese Siebold's beech (Hoshika, Haworth, et al., 2020; Watanabe et al., 2018) and Mediterranean oaks (Hoshika et al., 2022), although a similar reduction in *g*_m_ was not observed in European beech exposed to free‐air O_3_ exposure (Warren et al., 2007). However, there is a knowledge gap regarding how O_3_ affects *g*_m_ and its biological significance in the context of O_3_‐induced oxidative stress.

Mesophyll conductance is related to physical mesophyll structure and biochemical properties (Evans, 2021). After passing through the stomata, CO_2_ moves through the intercellular air spaces and dissolves in water within the cell wall. It then diffuses through the plasma membrane, diffuses over the cytoplasm, and finally reaches the stroma by crossing the chloroplast envelope. Mesophyll conductance can be influenced by environmental factors such as light (Busch et al., 2020) and temperature (Caemmerer & Evans, 2015). Flexas et al. (2008) summarised literature data showing that leaf mass per area (LMA) is a simple indicator to determine the potential *g*_m_. Mesophyll conductance is often correlated with the surface area of chloroplasts exposed to intercellular airspace (Evans et al., 1994). Hanba et al. (2004) reported that transgenic rice plants with aquaporin overexpression showed an enhancement of *g*_m_. Also, leaf N contributes to the activity of aquaporins or carbonic anhydrase relating to *g*_m_ (Buckley & Warren, 2014). In addition, some studies pointed out that abscisic acid (ABA) may induce a reduction of *g*_m_ (Brunetti et al., 2019; Mizokami et al., 2015, 2022; Sorrentino et al., 2016). We postulate that O_3_ may affect *g*_m_ because it alters mesophyll ultrastructure and thus leaf morphological parameters (Moura et al., 2018; Paoletti et al., 2009), modifies photosynthetic N use efficiency (PNUE) (Watanabe et al., 2013), and builds up ABA concentration in leaves (McAdam et al., 2017; Vainonen & Kangasjärvi, 2015). Several studies reported that the reduction of *g*_m_ under elevated O_3_ concentrations was negatively correlated with LMA (pubescent oak: Hoshika et al., 2022, poplars: Joffe et al., 2022), leaf density (Siebold's beech: Hoshika, Haworth, et al., 2020) and/or leaf ABA content (pubescent oak and pedunculate oak: Hoshika et al., 2022). However, LMA was not necessarily correlated with *g*_m_ in leaves exposed to O_3_ (Siebold's beech: Watanabe et al., 2018, poplars: Xu et al., 2019). Recently, Xu et al. (2023) and Joffe et al. (2024) reported that an O_3_‐induced reduction of *g*_m_ was correlated with increased cell wall thickness for poplars. However, as also mentioned in their paper, O_3_ effects on leaf ultrastructure are highly variable on both an intra‐ and inter‐specific basis. In fact, a reduction of *g*_m_ was found in O_3_‐exposed birch leaves (Eichelmann et al., 2004), although there was no change in cell wall thickness (Padu et al., 2005). A broad consensus has not yet been reached as to the mechanisms underlying *g*_m_ reduction under O_3_ exposure.

Poplars are light‐demanding trees widely used as a model species for plant physiology (Koike, 1995). We selected two poplar clones differing in O_3_ sensitivity: I‐214 (*Populus deltoides* W. Bartram ex Marshall × *P. nigra* L.) is less sensitive (Di Baccio et al., 2008) whereas Oxford (*P. maximowiczii* Henry × *P. berolinensis* Dippel) is very sensitive to O_3_ (Hoshika et al., 2018; Marzuoli et al., 2009; Zhang et al., 2018). Shoot growth of Oxford exhibits a rather ‘flushing‐like’ type, that is shoot development ceases in early August (Giovannelli et al., 2019; Zhang et al., 2018), while I‐214 continues shoot growth until the end of September (Giovannelli et al., 2007). The longer phenological development of I‐214 may have advantages for producing new leaves as a compensatory response against O_3_ damage during the growing season. Compensatory leaf growth due to O_3_ is often associated with accelerated leaf turnover (Pell et al., 1995). The new leaves formed under elevated O_3_ conditions may have different structural and physiological traits, which may affect *g*_m_ and then photosynthesis (Hartikainen et al., 2020). Therefore, we postulated that O_3_ effects on photosynthetic traits – especially *g*_m_ – may be dependent on leaf age, with poplar clones differing in leaf structural and physiological acclimation to growth under elevated O_3_ concentrations.

Our aim was to examine the relationship between leaf structural (LMA and anatomical parameters) and physiological parameters (water status and nitrogen, chlorophyll and ABA contents) with photosynthetic traits by employing a structural equation model (SEM) approach, which is a combined factorial and regression analytical method, allowing us to analyse the complex causal relationships among these variables (Fan et al., 2016).

## RESULTS

### Number of leaves and leaf longevity

**Figure 1:**
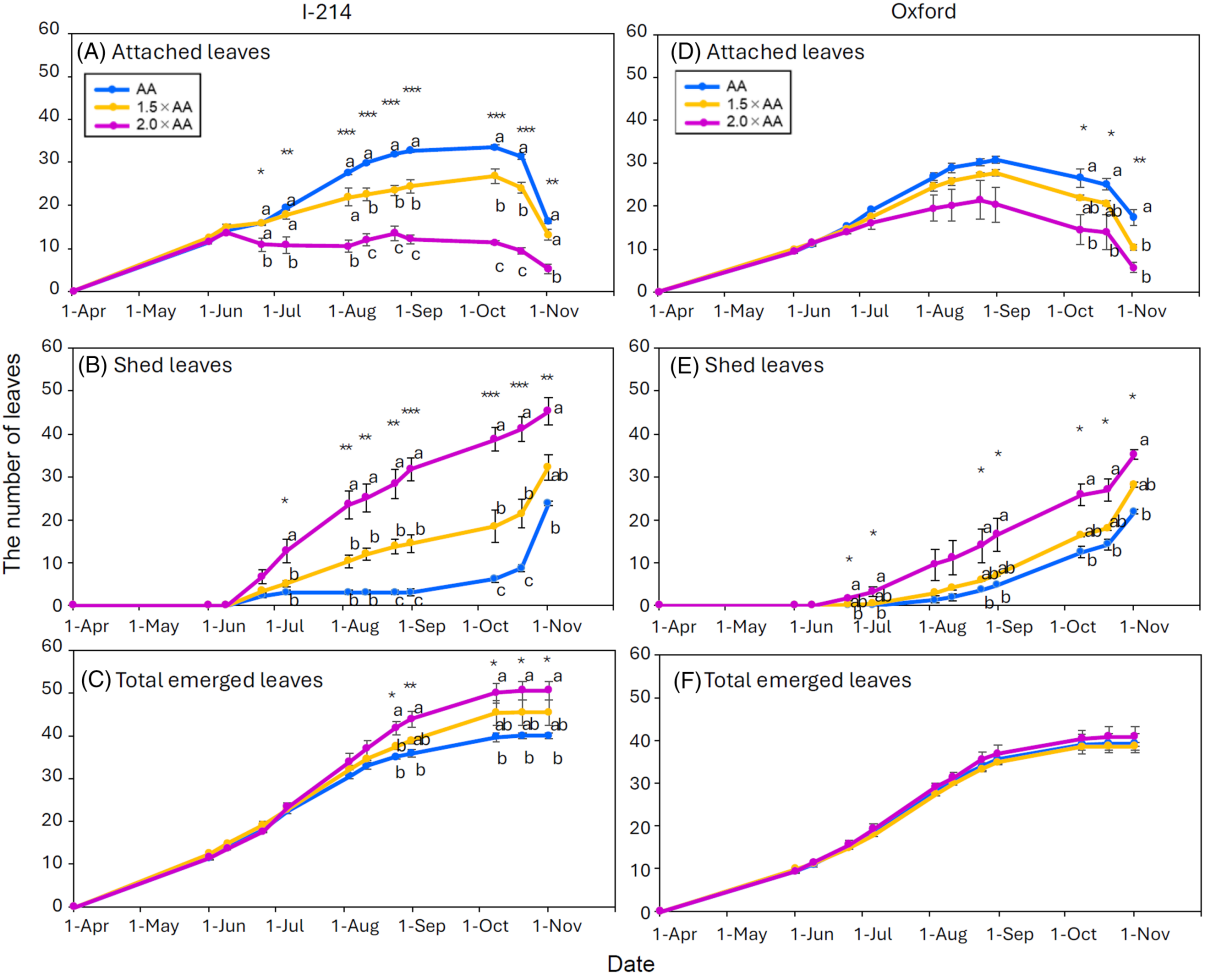
Number of attached leaves (A, D), shed leaves (B, E) and new leaf formation rate (C, F) at main shoots of I‐214 (A–C) and Oxford (D–F) poplar clones grown at three O_3_ levels (AA, ambient O_3_ concentration: blue points and blue line, 1.5 × AA: orange points and orange line, 2.0 × AA: violet points and violet line). Data are mean ± SE (*n* = 3 plots). Asterisks show the significance of anova: ***P ≤ 0.001, **P ≤ 0.01, *P ≤ 0.05. Different letters show significant differences among treatments (*P* ≤ 0.05, Tukey test) each measurement time. We did not find any significant difference among treatments for leaf formation rate in Oxford. The black arrow indicates a period for leaf gas exchange measurements and sampling for biochemistry and microscopic assessments.

A rapid increase in the number of shed leaves was observed in I‐214 after 36 days under 2.0 × AA O_3_ exposure (Figure 1B). On the other hand, the process of leaf shedding was relatively slow in O_3_‐exposed Oxford clones (Figure 1E). As a result, a statistically significant difference in the number of attached leaves among O_3_ treatments was found from mid‐June to October in I‐214 (Figure 1A), while Oxford showed differences among treatments only in October (Figure 1D). The new leaf formation rate did not show any difference among the O_3_ treatments in Oxford (Figure 1F), whereas O_3_ exposure enhanced the leaf formation rate due to an accelerated leaf turnover in I‐214 (Figure 1C). As a result, fumigation with O_3_ reduced the total leaf area for both poplar clones at the end of the experiment (Table S1).

Leaf longevity was approximately 100 to 109 days in both poplar clones under AA treatments (Table 1). However, longevity decreased with increasing O_3_ levels. Both enhanced O_3_ levels significantly decreased leaf longevity in I‐214 (1.5 × AA: −25%, 2.0 × AA: −62%) while a significant decrease was observed only in the highest level of O_3_ fumigation in Oxford (2.0 × AA: −31%).

**Table 1:** Leaf longevity of I‐214 and Oxford poplar clones grown at three O_3_ levels (AA, ambient O_3_ concentration, 1.5 × AA, 2.0 × AA)

Ozone treatments	Leaf longevity (days)	
	I‐214	Oxford
AA	108.4 ± 1.3a	99.7 ± 2.7ab
1.5 × AA	81.2 ± 7.6bc	91.1 ± 2.0abc
2.0 × AA	41.3 ± 5.0d	68.4 ± 7.3c
anova results		
O_3_	***	
Clone	*	
O_3_ × Clone	*	

Data are mean ± SE (*n* = 3 plots). Asterisks show the significance of anova: ***P ≤ 0.001, *P ≤ 0.05. Different letters show significant differences among the combination of treatments and clones (*P* ≤ 0.05, Tukey test).

### Ozone and age effects on gas exchange traits of leaves

**Figure 2:**
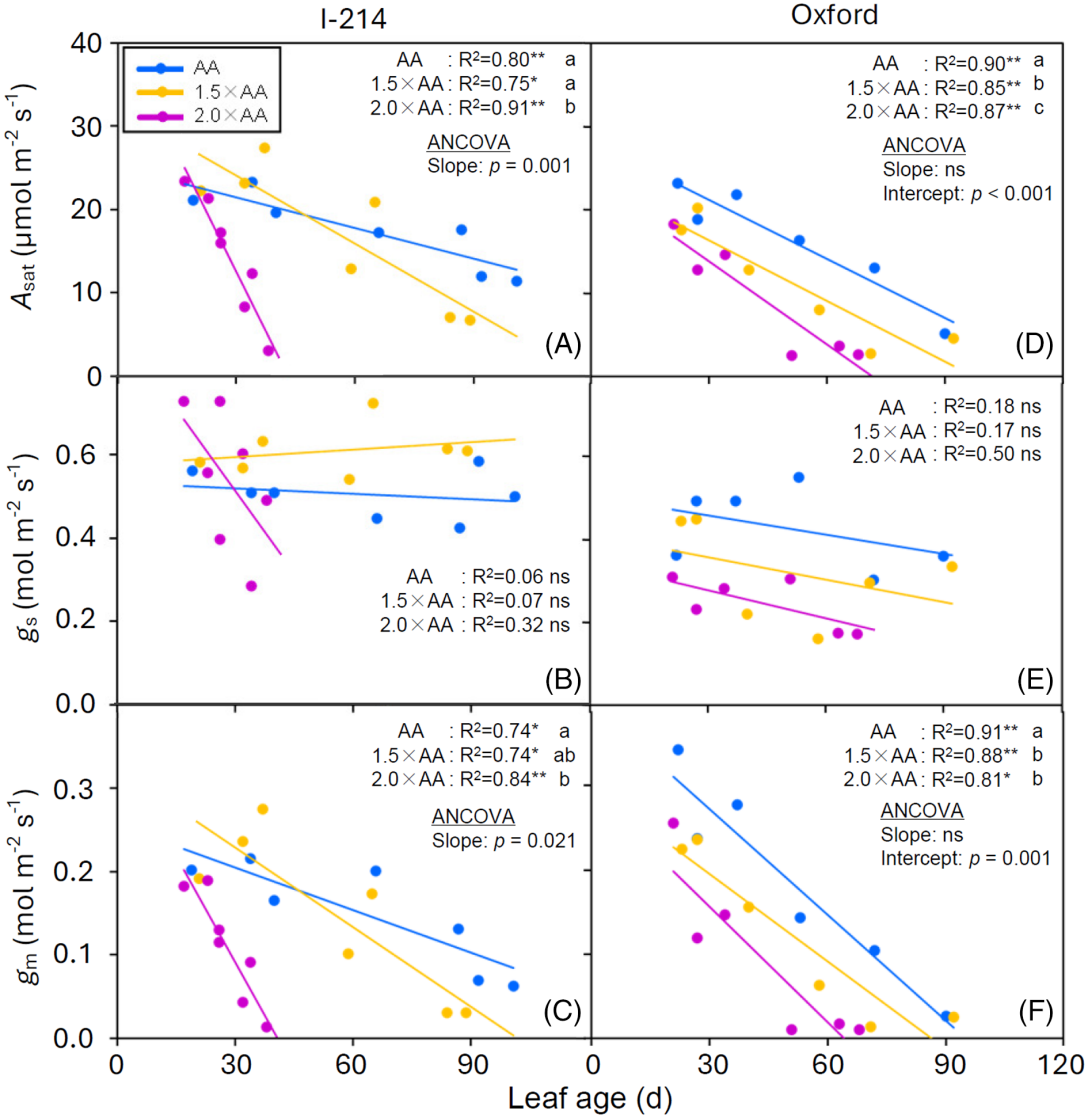
Relationships between light‐saturated net photosynthetic rate [*A*_sat_] (A, D), stomatal conductance [*g*_s_] (B, E) or mesophyll conductance [*g*_m_] (C, F) and leaf age in I‐214 (A–C) and Oxford (D–F) poplar clones grown at three O_3_ levels (AA, ambient O_3_ concentration: blue points and blue line, 1.5 × AA: orange points and orange line, 2.0 × AA: violet points and violet line) (*n* = 3 plants). Linear regression analysis: **P ≤ 0.01, *P ≤ 0.05, ns denotes not significant. When at least two regression lines were statistically significant, ancova was applied to test for significant differences of the regressions among O_3_ treatments. Different letters denote significant differences of the regressions between O_3_ treatments (*P* ≤ 0.05).

Net photosynthetic rate decreased with increasing leaf age in both clones (Figure 2A,D). A rapid decrease in *A*_sat_ with leaf ageing was found in I‐214 grown under 2.0 × AA exposure, as confirmed by the significantly different slope of the regressions among O_3_ treatments. In Oxford, O_3_ caused a significant difference in the intercept of the relationships between *A*_sat_ and leaf age, indicating that an O_3_‐induced decrease in *A*_sat_ occurred even in young leaves. Accordingly, most photosynthetic parameters (*g*_m_, *V*_cmax_, *J*_max_, *R*_pr_, ΦPSII) followed a similar tendency in relation to leaf ageing (Figure 2C,F; Figures S2 and S3c,f). However, *g*_s_ and *R*_n_ did not show such a clear relationship with leaf ageing (Figure 2B,E; Figure S3b,e). Although there was no dependency of *g*_s_ on leaf age, we found a significant decrease in the *g*_s_ of I‐214 with 2.0 × AA O_3_ exposure (−42% in 2.0 × AA compared to AA, anova: *P* = 0.016). Interestingly, we found that there was a different response in *g*_s_ during nighttime between the two clones. Stomatal conductance at night (*g*_night_) increased with increasing leaf age in the two enhanced O_3_ treatments in Oxford (Figure S3d). I‐214 did not show such a trend (Figure S3a), and O_3_ treatments significantly increased *g*_night_ irrespective of the leaf ageing process (+44% in 1.5 × AA and +286% in 2.0 × AA compared to AA, anova: *p* = 0.001).

**Figure 3:**
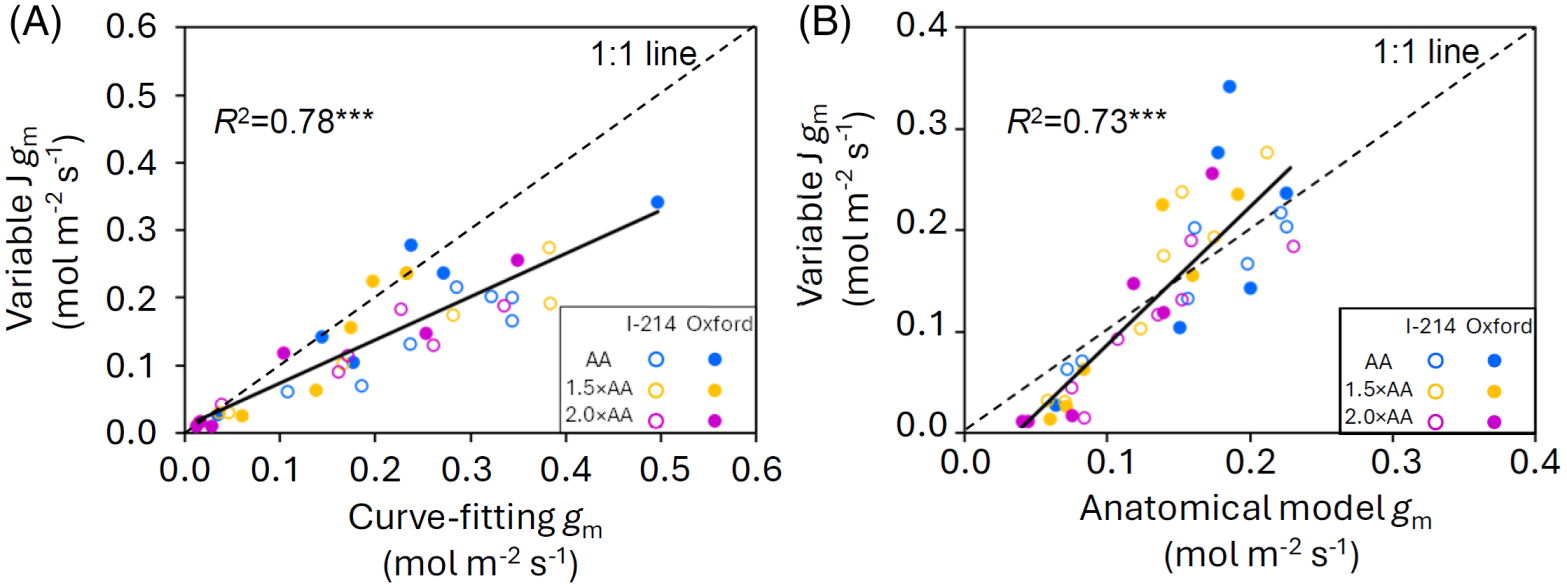
Relationship between mesophyll conductance (*g*_m_) values derived from the variable J method and the *A*/*C*_i_ curve fitting (A) and the anatomy‐derived method (B) in I‐214 (empty circle) and Oxford (closed circle) poplar clones grown at three O_3_ levels (AA, ambient O_3_ concentration: blue points, 1.5 × AA : orange points, 2.0 × AA: violet points) (*n* = 3 plants). A linear regression analysis: ***P ≤ 0.001.

**Figure 4:**
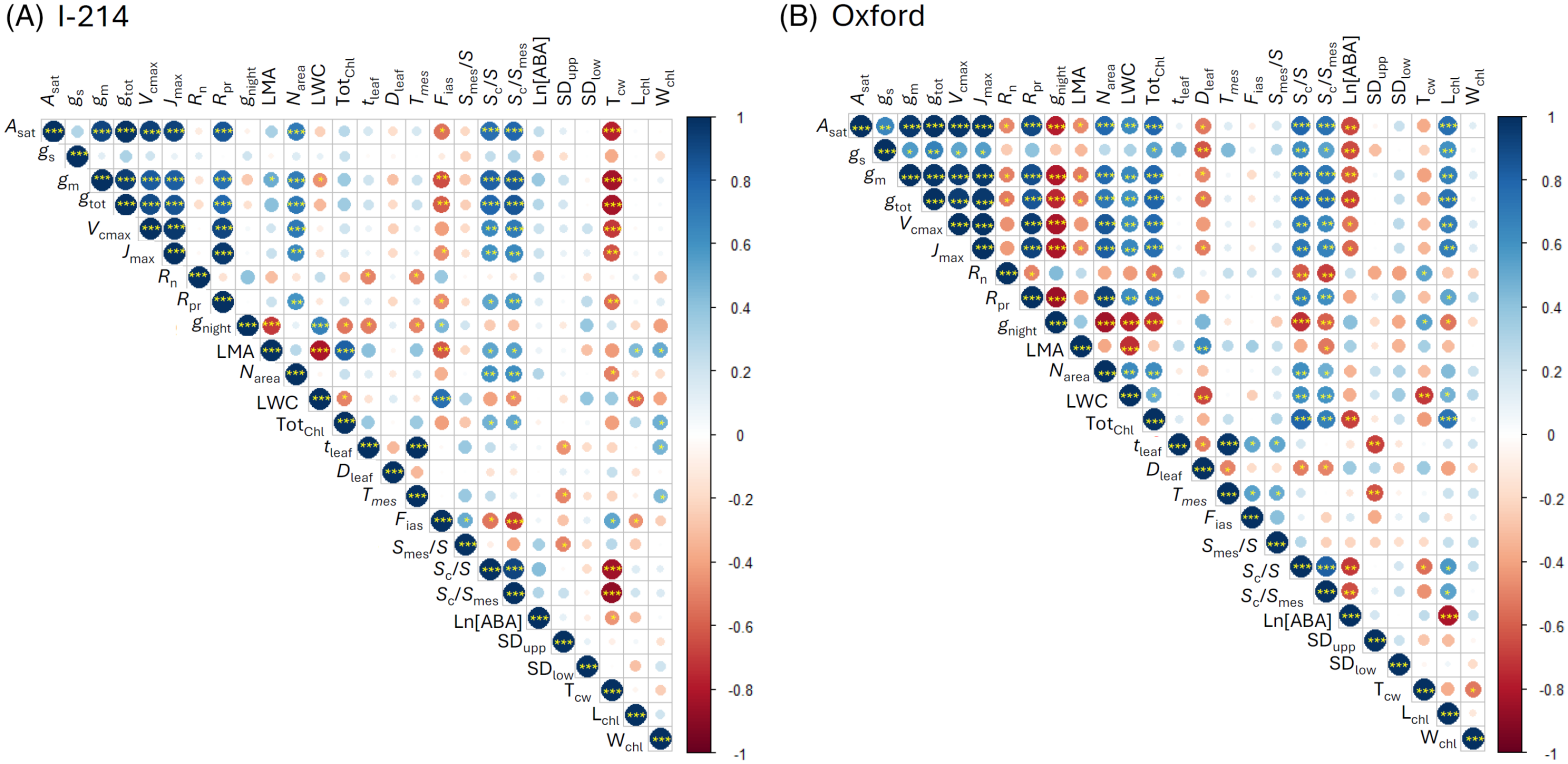
Correlation matrix of leaf structural, physiological and photosynthetic parameters in I‐214 (A) and Oxford poplar clones (B) grown at three O_3_ levels (AA, ambient O_3_ concentration, 1.5 × AA, 2.0 × AA). Spearman analysis: ***P ≤ 0.001, **P ≤ 0.01, *P ≤ 0.05. Blue denotes a positive correlation while red denotes a negative correlation.

**Figure 5:**
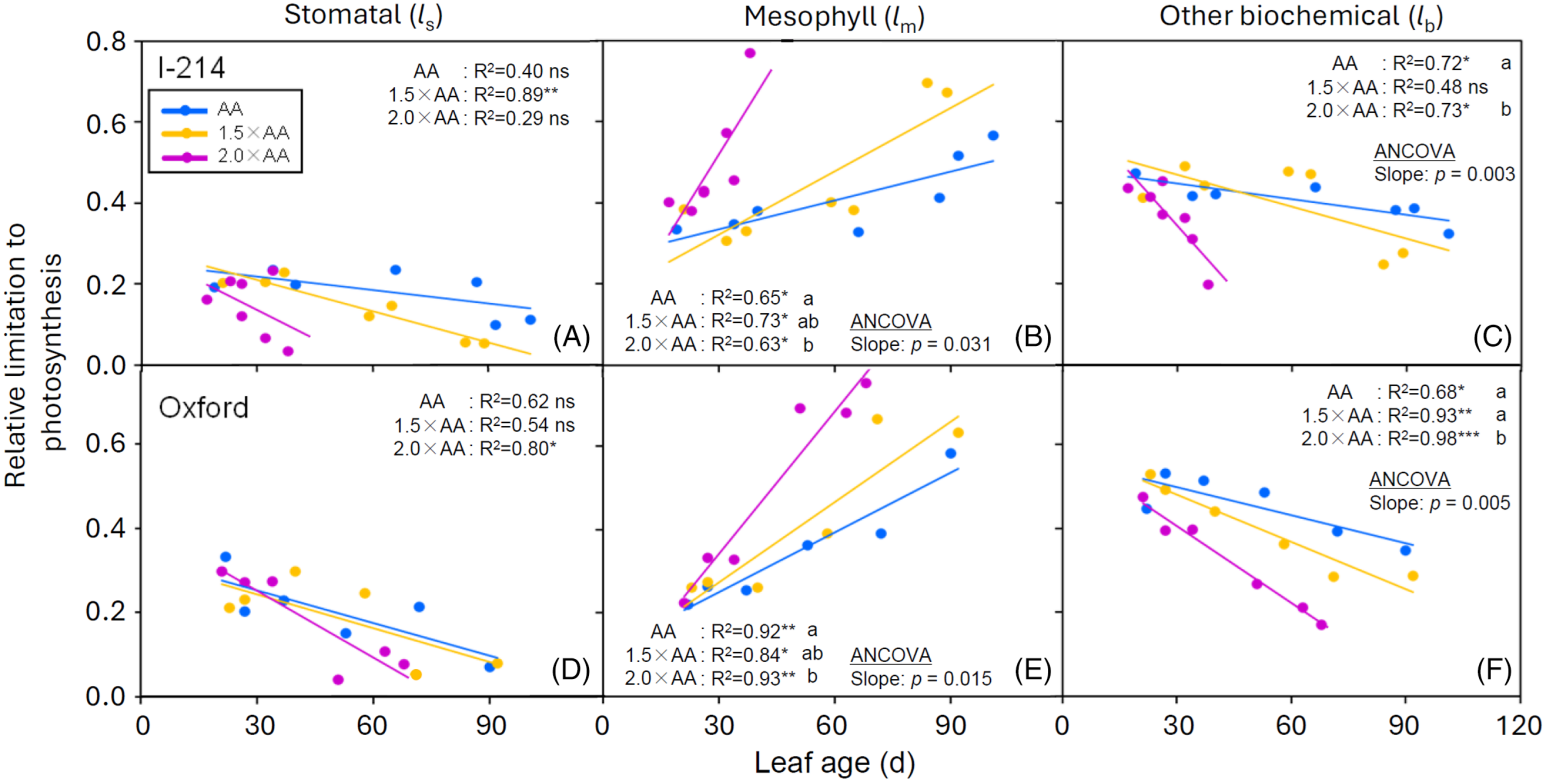
Relationships between the relative contributions of stomatal [*l*_s_] (A, D), mesophyll conductance [*l*_m_] (B, E) and other biochemical limitations [*l*_b_] (C, F) to light‐saturated net photosynthesis [*A*_sat_] and leaf age in I‐214 (A–C) and Oxford (D–F) poplar clones grown at three O_3_ levels (AA, ambient O_3_ concentration: blue points and blue line, 1.5 × AA: orange points and orange line, 2.0 × AA: violet points and violet line) (*n* = 3 plants). Linear regression analysis: ***P ≤ 0.001, **P ≤ 0.01, *P ≤ 0.05, ns denotes not significant. When at least two regression lines were statistically significant, ancova was applied to test for significant differences of the regressions among O_3_ treatments. Different letters denote significant differences of the regressions among O_3_ treatments (*P* ≤ 0.05).

The *g*_m_ values derived from the fitting *A*/*C*_i_ curve approach and anatomical modelling were consistent with those calculated from the variable J method, although the curve‐fitting *g*_m_ tended to be higher than the variable J‐derived *g*_m_ (Figure 3). The light‐saturated net photosynthetic rate was positively correlated to *g*_m_ and *g*_tot_ in both clones, but not significantly to *g*_s_ in I‐214 (Figure 4; Figure S4). The correlation between *A*_sat_ and *g*_tot_ was largest among the diffusive parameters for both clones. This was also confirmed by the Grassi and Magnani limitation analysis, suggesting that *g*_m_ showed a relatively high contribution to photosynthesis, especially under 2.0 × AA regardless of the poplar clones (Figure 5).

### Ozone and age effects on leaf nitrogen, water, chlorophyll and abscisic acid content

Leaf N content per unit area decreased with increasing leaf age in both clones (Figure S5a,d), although the relationship between N_area_ and leaf age in I‐214 under AA conditions was not significant. It should be noted that I‐214 grown under 1.5 × AA and 2.0 × AA conditions had a relatively high N_area_ in young leaves (about 1.5 g m^−2^), and a rapid decrease in N_area_ was shown as leaves aged, especially in 2.0 × AA treatments. There were significant relationships between Tot_Chl_ and leaf age in 2.0 × AA for I‐214 and in all O_3_ treatments for Oxford (Figure S5b–e). The content of ABA increased with increasing leaf age in I‐214 under 2.0 × AA conditions (Figure S5c). Conversely, ABA content in I‐214 decreased with leaf age under AA and 1.5 × AA conditions. A positive relationship between ABA and leaf age was observed in Oxford under 1.5 × AA and 2.0 × AA conditions (Figure S5f). In addition, a higher leaf ABA concentration was found in Oxford under 2.0 × AA conditions, as indicated by a significant increase in the intercept of the regression. A relatively low PNUE and *V*_cmax_/N_area_ were observed under 2.0 × AA conditions in both poplar clones (Figure S6). In terms of LWC, it seems that young leaves under 2.0 × AA conditions had a relatively high value in I‐214, although O_3_ effects were not clear in Oxford (Figure S7b,e).

### Ozone and age effects on leaf structural parameters

There was no clear age dependency on most structural parameters (LMA, *D*_leaf_, *t*_leaf_, *T*_mes_, *F*_ias_, *S*_mes_/*S*, *W*_chl_, *SD*_upp_, *SD*_low_) in both clones (Figures S7–S11; Tables S3 and S4). There appeared to be relatively high *F*_ias_ in O_3_‐exposed I‐214 leaves (Figure S8c), although O_3_ effects on this parameter were not statistically significant (anova: *P* = 0.056). Leaf ageing negatively affected *S*_c_/*S* and *S*_c_/*S*_mes_, especially in I‐214 (Figure S8b,c). In addition, ancova revealed that O_3_ significantly affected relationships between *S*_c_/*S* or *S*_c_/*S*_mes_ and leaf age, indicating that O_3_ accelerated the ageing process in this clone. Interestingly, *S*_c_/*S*_mes_ seemed to be enhanced in young leaves of I‐214 under 1.5 × AA (Figure S9c). Such a positive trend was also observed in Oxford under 1.5 × AA regardless of the leaf ageing process (+14% and +72% in 1.5 × AA compared to AA and 2.0 × AA, respectively; anova: *P* = 0.044). A clear age dependency on *T*_cw_ was found in I‐214, and O_3_ increased *T*_cw_, especially under 2.0 × AA (Figure S10a). However, such a trend was not observed in Oxford. For *L*_chl_, a negative effect of O_3_ was found to be particularly evident in Oxford poplars (Figure S10e).

**Figure 6:**
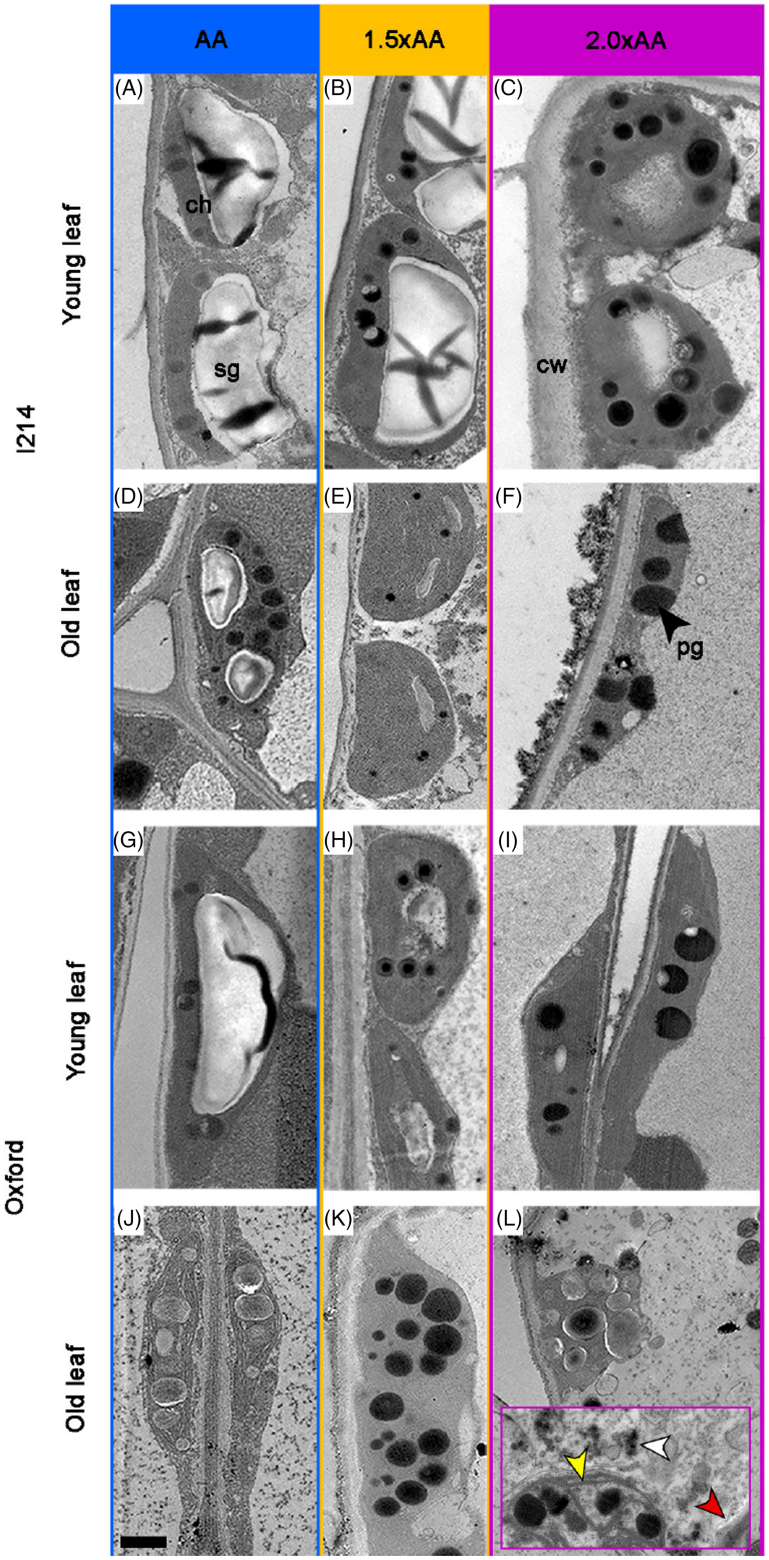
Transmission electron microscopy leaf cross‐sections of young and old leaves for I‐214 (A–F) and Oxford (G–L) poplar clones grown at three O_3_ levels (AA, ambient O_3_ concentration: blue frame, 1.5 × AA: orange frame, 2.0 × AA: violet frame). Leaf age of young leaves, I‐214: 19 days in AA, 21 days in 1.5 × AA and 17 days in 2.0 × AA; Oxford: 22 days in AA, 23 days in 1.5 × AA and 21 days in 2.0 × AA. Leaf age of old leaves, I‐214: 101 days in AA, 89 days in 1.5 × AA and 38 days in 2.0 × AA; Oxford: 90 days in AA, 92 days in 1.5 × AA and 68 days in 2.0 × AA. ch: chloroplast; pg: plastoglobuli; cw: cell wall; sg: starch grains. In the purple frame of (L), white arrow: cell death‐associated bodies; red arrow: cellular shrinkage; yellow arrow: chloroplast breakdown. Bars in (J) are valid for all the figures = 1 μm.

These results were confirmed by microscopic assessments. Figure 6 and Figure S13 show light and TEM micrographs, respectively, of cross‐sections of young (19–23 days) and old leaves (38–101 days). Mesophyll tissue integrity was observed in new leaves of both clones (Figure 6A,C,G,I; Figure S14a,c,g,i), especially in samples from the AA treatment where the accumulation of starch grains was observed (detail in Figure S13g). In the 2.0 × AA samples, clear signs of chloroplast degradation were observed, including an increased number of plastoglobuli and a greater proportion of chloroplast areas occupied by these structures, particularly in young leaves (Figure 6C versus Figure 6A and Figure 6I versus Figure 6G). Notably, in the Oxford clone, cell death‐associated bodies (Figure 6L, detail – white arrow), cellular shrinkage (Figure 6L, detail – red arrow) and chloroplast breakdown (Figure 6L, detail – yellow arrow) were evident, suggesting internal fragmentation and indicating the progression of programmed cell death. Interestingly, relatively thick cell walls were found in young I‐214 leaves under 2.0 × AA (Figure 6C). In the older leaves of I‐214 (Figure 6D–F; Figure S13d–f), we found disrupted cytoplasm and a condensation of remnants within cell death‐associated bodies – a hallmark of programmed cell death, resulting in chloroplast deformation and a decreased amount of starch grains as a consequence of lower photosynthesis. It should be noted that structural marks of oxidative stress due to O_3_ exposure were observed at 1.5 × AA and 2.0 × AA, but not in AA samples. In fact, those old leaves exposed to elevated O_3_ concentration exhibited a hypersensitive response (HR‐like) as discretely distributed, disrupted and collapsed palisade parenchyma cells. On the other hand, old leaves from Oxford apparently showed similar structural degradation among the O_3_ treatments (Figure 6J–L; Figure S13j–l), but the HR‐like process was observed only in 2.0 × AA samples. Chloroplast degradation marks were intense for this clone.

### Causal relationships between leaf structural or physiological parameters and mesophyll conductance were identified by the use of a structural equation model (SEM)

**Figure 7:**
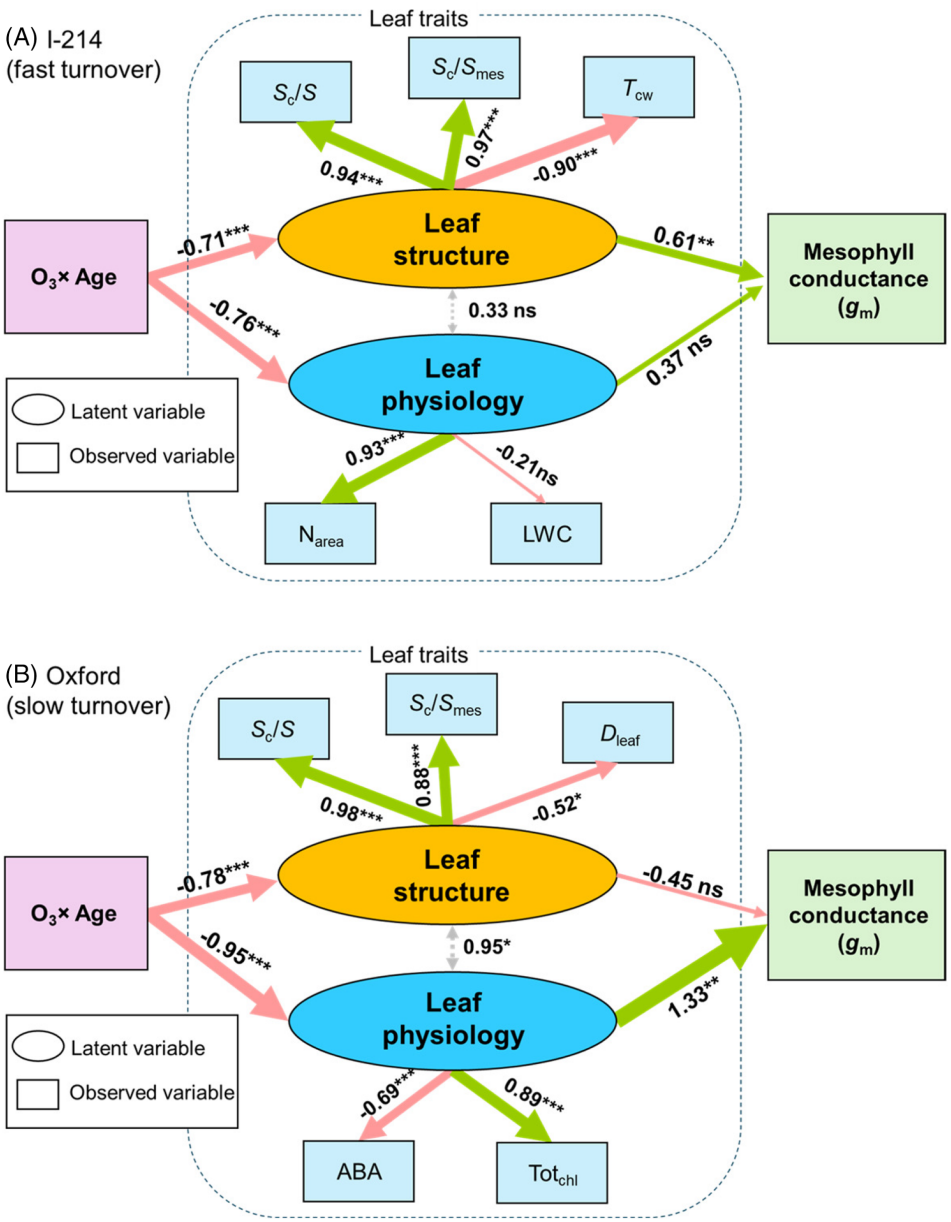
Structural equation modelling (SEM) for the O_3_ × leaf age interaction on mesophyll CO_2_ diffusion considering causal relationships between leaf structure or physiology with mesophyll conductance [*g*_m_]. Two latent variables were finally used: leaf structure (characterised by *S*_c_/*S*, *S*_c_/*S*_mes_, *T*_cw_ for I‐214 and by *S*_c_/*S*, *S*_c_/*S*_mes_, *D*_leaf_ for Oxford) and leaf physiology (characterised by N_area_, LWC for I‐214 and ABA, Tot_Chl_ for Oxford). Single‐headed arrows indicate causal relationships, showing that one variable directly affects another. Negative paths are shown by red lines while positive paths are shown by green lines. Double‐headed grey arrows represent correlations between latent variables. The values of the arrows denote the standardised SEM path coefficients indicating the magnitude of the strength of causality (*z*‐test: ***P ≤ 0.001, **P ≤ 0.01, *P ≤ 0.05, ns denotes not significant, *N* = 21 for I‐214, *N* = 18 for Oxford). The performance of the models is specified in Table S2.

We constructed a SEM framework to investigate the O_3_ × leaf age interaction on mesophyll CO_2_ diffusion considering causal relationships among leaf structural, physiological parameters and *g*_m_ (Figure 7). According to the correlation matrix, *g*_m_ was significantly correlated with several structural (LMA, *F*_ias_, *S*_c_/*S*, *S*_c_/*S*_mes_ and *T*_cw_) and physiological parameters (N_area_, LWC) in I‐214, while significant correlations were found in Oxford with similar but different leaf structural (LMA, *D*_leaf_, *S*_c_/*S*, *S*_c_/*S*_mes_ and *L*_chl_) and physiological parameters (N_area_, ABA, Tot_Chl_, LWC) (Figure 4). Among them, three leaf structural (*S*_c_/*S*, *S*_c_/*S*_mes_, *T*_cw_) and two physiological variables (N_area_, LWC) were selected for the best model for I‐214, and three leaf structural (*S*_c_/*S*, *S*_c_/*S*_mes_, *D*_leaf_) and two physiological variables (ABA, Tot_Chl_) were selected for Oxford (Figure 7; Table S2).

The SEMs indicated that the interaction of O_3_ and leaf age significantly affected leaf structural and physiological variables for both clones. In I‐214, *g*_m_ was significantly influenced by leaf structural but not by physiological parameters. However, in Oxford, the opposite result was found, and *g*_m_ was mainly affected by leaf physiological parameters such as ABA and Tot_Chl_. These results were consistent but not solely observed within the correlation metrics (Figure 4) or regression analyses (Figure S12).

## DISCUSSION

Leaf longevity was not statistically different between the two poplar clones in ambient air. However, I‐214 shifted to a rapid leaf turnover under elevated O_3_ conditions, forming new productive leaves, while Oxford showed a rather ‘conservative’ response, maintaining injured leaves as long as possible. Such a discrepancy in leaf development as a stress‐related response between species may be related to a different phenological pattern, that is flushing and successive leaf developmental types (Hoshika, Tatsuda, et al., 2013; Manninen et al., 2009). Ozone exposure differentially influenced the age‐related variations in photosynthetic capacity of the two poplar clones in the present study through their leaf developmental response and premature leaf senescence.

To assess *g*_m_, three methods were applied. The anatomical method may not be able to express short‐term physiological changes such as the deactivation of RubisCO enzymes. However, we found an agreement between the anatomical and the variable J derived *g*_m_ values. This result indicates that the physiologically active parts somehow function, although chloroplasts were gradually degrading after exposure to O_3_. For the variable J method, a standard value for leaf absorptance (*α* = 0.85, Protocol S1) was used, which might lead to an overestimation of ETR, resulting in an underestimation of *g*_m_. However, the effect seemed to be minor (9.4% change as an average for old leaves, data not shown, when applying *α* = 0.75 as a case of chlorophyll degradated old poplar leaves, Bauerle et al., 2004), which agrees with a sensitivity analysis by Gilbert et al. (2012) where no significant difference in calculated *g*_m_ values was found between measured *α* and the standard *α* of 0.85. Although the *g*_m_ values derived from three methods were consistent with each other, further study will be recommended to examine a systematic bias of the methodology for the calculation of *g*_m_.

In addition, SEM allowed us to improve our understanding of the causal relationships among variables when considering the interactive effects of O_3_ and leaf age; thus, developing a conceptual framework of leaf‐level physiological and structural acclimation against O_3_ stress. As a result, *g*_m_ in the I‐214 poplar clone with ‘active’ leaf growth was mainly influenced by leaf structural parameters such as *S*_c_/*S* and *T*_cw_ under elevated O_3_ conditions, whereas leaf physiological parameters such as ABA affected *g*_m_ in the Oxford poplar clone with a ‘conservative’ leaf development. In the following section, we describe several important points of leaf‐level physiological and structural acclimation relating to photosynthesis by comparing the response to O_3_ between the two poplar clones.

### Leaf anatomy and mesophyll conductance

Ozone and its secondary formed reactive oxygen species (ROS) often disrupt the physical integrity of mesophyll cells, modifying leaf ultra‐structures such as cell walls, which is likely to affect the diffusion of CO_2_ inside leaves (e.g. Moura et al., 2018). Xu et al. (2023) and Joffe et al. (2024) recently suggested that an increase in *T*_cw_ may negatively affect *g*_m_ under elevated O_3_ conditions. Our results in I‐214 poplars were in agreement with their results. Microscopic analyses suggested that cell walls under 2.0 × AA were relatively thick even in young leaves for this poplar clone as an acclimation response, which enabled physical enhancement of the resistance against O_3_ stress (Feng et al., 2018; Moldau et al., 1998). However, this may also lead to an increase in the CO_2_ diffusion path length in the mesophyll and result in a reduction in photosynthesis. Mesophyll conductance reflects the biochemical and physical properties of the mesophyll affecting CO_2_ diffusion (Flexas et al., 2008). Mesophyll conductance can be viewed as a flux‐weighted parameter reflecting the availability of CO_2_ in the sub‐stomatal air‐spaces, the consumption of CO_2_ in the chloroplast, and the capacity for CO_2_ transport across the mesophyll (Tholen et al., 2012). Ozone‐exposed leaves in I‐214 appeared to show a higher intercellular air space, especially in old leaves, which may have resulted in enhanced CO_2_ diffusion in the gas phase. However, this was not a determinant factor in the regulation of *g*_m_ in the target poplars under elevated O_3_ conditions as confirmed by SEM. In old leaves, chloroplast frequency was markedly decreased in association with a collapse of mesophyll cells. Therefore, the negative effect of O_3_ on *g*_m_ was mainly associated with a restriction in liquid phase CO_2_ diffusion in mesophyll layers rather than gas phase CO_2_ diffusion. Also, in old I‐214 leaves, we observed potential starch accumulation in the microscopic analyses. The accumulation of starch grains within chloroplasts might impede CO_2_ diffusion in the liquid phase because chloroplasts become thicker due to the storage of starch (Nafziger & Koller, 1976; Sawada et al., 2001). However, we did not find such increases in chloroplast thickness in the I‐214 clones.

In the Oxford poplar clones, a reduction of *g*_m_ was not explained by a variation of *T*_cw_. The Oxford clone showed an enhancement of *S*_c_/*S*_mes_ in 1.5 × AA. Although this acclimation response may have led to a relatively high *g*_m_ to maintain carbon assimilation rates, there was not a positive effect on *g*_m_ due to the possible negative effect of leaf ABA on this parameter.

### Effect of leaf ABA on photosynthesis

Once O_3_ enters a leaf, it generates ROS, which act as messengers in ABA metabolism (Vainonen & Kangasjärvi, 2015). Therefore, leaf ABA concentrations may be elevated under high O_3_ conditions and cause a reduction in both *g*_s_ and *g*_m_, as reported in Mediterranean oak species (Hoshika et al., 2022). In fact, this process was confirmed in Oxford. As the SEM demonstrated, a reduction of *g*_m_ in Oxford was causally related to ABA rather than leaf structural parameters. It seems that elevated leaf ABA concentrations offset the positive effect of enhanced *S*_c_/*S*_mes_ on *g*_m_ under a moderate level of O_3_ exposure (1.5 × AA). The physiological mechanisms of the effect of ABA on *g*_m_ are not clear. In Oxford, ABA seems to be involved in a signaling response to modulate stomatal function and photosynthesis to avoid damage under moderate and elevated O_3_ concentrations (with proline and sugars; Pisuttu et al., 2024). These physiochemical responses might be a part of programmed cell death and a plastic trait of leaves under stress conditions (Cotrozzi et al., 2017; Marchica et al., 2022), where the interrelation between leaf structure and physiology, such as ABA, was confirmed by the SEM analysis. However, this orchestrated signaling response did not counteract some disorders in terms of photosynthetic performance in older leaves of Oxford. ABA might also affect the membranes through reduced aquaporin activity, which is accompanied by a reduction in *g*_s_, thus limiting CO_2_ transport across the mesophyll to the chloroplast (Sorrentino et al., 2016). It is therefore worthwhile to investigate potential changes in the status of aquaporins in O_3_‐exposed leaves in a future study.

There was no significant effect of ABA on *g*_m_ in O_3_‐exposed I‐214 leaves. In fact, leaf ABA concentration did not increase significantly during the whole period under elevated O_3_ treatments. It is worth noting that the elevated O_3_ concentrations induced multiple signals that might be part of a premature leaf senescence process (Sharma & Verslues, 2010). In particular, under 2.0 × AA conditions, the content of ABA increased due to leaf ageing, relating to a premature leaf senescence process and a faster leaf turnover (Pisuttu et al., 2024).

### Leaf nitrogen allocation and photosynthesis

Leaf N is also considered an essential factor in determining photosynthetic capacity (Larcher, 2003). In fact, leaf N may serve as a key element in protein synthesis and is needed for enzyme abundance and activity such as RubisCO, aquaporins or carbonic anhydrase relating to *g*_m_ (Buckley & Warren, 2014). Damaged leaves under elevated O_3_ conditions showed a low photosynthetic capacity with increasing ‘costs’ for maintenance rather than ‘benefits’ for production (Chapin et al., 1980; Villar et al., 2021). Therefore, N remobilisation from damaged leaves to expanding new young leaves through accelerated leaf turnover may be beneficial to maintain carbon assimilation rates under elevated O_3_ concentrations (Kitao et al., 2015; Pell et al., 1995). Such a response occurred in I‐214, in which a relatively high leaf N content was found in new leaves formed under 1.5 × AA and 2.0 × AA treatments. However, a positive effect of high leaf N concentration on photosynthetic traits such as *g*_m_ was limited in 2.0 × AA due to less efficient use of N for photosynthesis (i.e. PNUE). As indicated by a low *V*_cmax_/N_area_ in elevated O_3_ treatments, this may be due to a reduced N allocation to photosynthetic proteins as reported in Japanese Siebold's beech (Watanabe et al., 2013) and hybrid poplars (Shang et al., 2019).

Forming new leaves requires resources from plants, although O_3_ may impair the resorption of N from old leaves because N released from degraded chloroplasts cannot be re‐used by N resorption (Shi et al., 2017; Uddling et al., 2006). Nocturnal water flux caused by stomatal opening at night may have a potential role in nutrient acquisition by roots especially for mobile nutrients such as N (de Dios et al., 2013; Eller et al., 2017). Interestingly, 2.0 × AA O_3_ exposure enhanced *g*_night_ in I‐214 regardless of leaf age. Therefore, an elevated nocturnal water flux was expected in those plants, and it may have driven root N uptake. Such extra N would support the high leaf turnover found in I‐214 exposed to O_3_. However, little is known about the potential biological significance of enhanced *g*_night_ due to O_3_ exposure (Hoshika et al., 2019).

In summary, as we hypothesized, O_3_ affected the age‐related variation of photosynthesis, and different causal mechanisms were found between the two poplar clones differing in phenological responses under elevated O_3_ concentrations: I‐214 showed ‘active’ new leaf forming pattern under elevated O_3_ concentrations, whereas Oxford showed a ‘conservative’ response by maintaining old or injured leaves. Mesophyll conductance was closely related to the variation of photosynthesis in both poplar clones. In the I‐214, with the fast leaf turnover, the mesophyll CO_2_ diffusion was affected mainly by leaf structure (*S*_c_/*S*, *S*_c_/*S*_mes_, *T*_cw_), which was enhanced in new leaves to potentially contribute to the resistance capacity against O_3_ stress. However, a rapid decrease in chloroplast frequency with leaf age was also observed prior to a decrease in photosynthetic rate, especially in 2.0 × AA O_3_ exposed leaves. On the other hand, *g*_m_ was affected by leaf physiological factors – with a marked negative effect of ABA – rather than structural parameters in the Oxford clones, showing slow leaf turnover.

Since in this study only two clones were examined as representatives with different phenological patterns, it is recommended that future studies examine other poplar clones to extend our knowledge of the effects of O_3_ on *g*_m_. Nonetheless, our results demonstrated that the contribution of *g*_m_ to the photosynthetic decline in poplars under elevated O_3_ conditions was relatively high; in fact, *g*_m_ was not considered in previous modeling studies (Lombardozzi et al., 2015; Sitch et al., 2007). Future studies on the effects of O_3_ on *g*_m_ for various species will be indispensable for modeling, allowing improved estimation of plant carbon sequestration under future O_3_ pollution and climate change.

## MATERIALS AND METHODS

### Experimental site and plant material

Experiments were conducted in an experimental garden at Sesto Fiorentino, Central Italy (43°49′ N, 11°12′ E, 55 m a.s.l.). Cuttings of Oxford and I‐214 poplar clones of uniform size (approximately 15 cm) were prepared in winter 2019 and planted into 10 L plastic pots filled with sand:peat:soil (1:1:1, v:v:v). In 2020, similar‐size rooted cuttings were exposed to three O_3_ treatments (AA, ambient O_3_ concentration; 1.5 × AA, 1.5 times ambient O_3_ concentration; 2.0 × AA, twice ambient O_3_ concentration) from 20 May to 31 October in a Free‐Air Controlled Exposure (FACE) facility. The production of O_3_ was achieved from pure oxygen with an O_3_ generator (Mod. TOGC13X, Triogen Ltd., Glasgow, Scotland), and the O_3_ enriched air was delivered for fumigation with vertical Teflon tubing. The technical details of the O_3_ FACE can be found in Paoletti et al. (2017). Daily mean hourly O_3_ concentration over the experimental period was 37.5 nmol mol^−1^ at AA, 52.3 nmol mol^−1^ at 1.5 × AA, and 73.3 nmol mol^−1^ at 2.0 × AA. These O_3_ levels are consistent with the levels observed in polluted areas of the Northern Hemisphere (Mills et al., 2018). We assigned three replicated plots (*L* × *W* × *H*: 5 × 5 × 2 m) to each O_3_ treatment containing four plants per clone (36 plants per each clone, in total). All plants were supplied with water every day to maintain substrate moisture close to field capacity (~0.295 m^3^ m^−3^, Paoletti et al., 2017).

### Measurement of leaf phenology

The number of leaves at the main shoot was counted at 2‐ to 3‐week intervals during the experimental period. In parallel, the number of shed leaves was examined by counting the leaf traces. Using this phenological dataset, potential leaf longevity was estimated according to Kikuzawa and Lechowicz (2011) as follows:

(1) $$\mathrm{LL}=\frac{\mathrm{NAL}\times \mathrm{LD}}{\text{NTEL}},$$

where NAL is the mean number of attached leaves, LD is the leaf duration, and NTEL is the mean number of total emerged leaves. The new leaf formation rate was calculated according to the difference in the number of total emerged leaves between two survey dates. We also checked and marked new leaves every 3 days (thus the potential error of calculated leaf age was ±3 days) and selected fully expanded target leaves for gas exchange measurements (Leaf age >14 days in I‐214 and 18 days in Oxford, according to the leaf length measurement, see Figure S1).

### Measurement of leaf gas exchange

Concurrent measurements of leaf gas exchange and chlorophyll fluorescence were conducted in fully expanded leaves differing in age (two to three differently‐aged leaves per three plants per clone per treatment) using a portable infra‐red photosynthesis measurement system (Model LI‐6800; Li‐Cor Instruments, Lincoln, NE, USA) from 26 August to 11 September 2020 (clear sky days, 9:00 h to 12:00 h). First, we determined the light saturated net photosynthetic rate (*A*_sat_) and stomatal conductance (*g*_s_) at the ambient concentration of CO_2_ (400 μmol mol^−1^), a photosynthetic photon flux density (PPFD) of 1500 μmol^−2^ m^−2^ sec^−1^ with an LED light source (10% blue and 90% red light, Flexas et al., 2022), a leaf temperature of 25°C and relative humidity (RH) of 50%. After that, to assess photosynthetic capacity, the response of leaf net assimilation rate (*A*) to the sub‐stomatal CO_2_ concentration (*C*_i_), that is the *A*/*C*_i_ curve, was measured over 12 CO_2_ steps (*C*_a_: 25, 50, 100, 200, 300, 400, 600, 800, 1100, 1400, 1700, 2000 μmol mol^−1^). Each measurement step in the *A*/*C*_i_ curve took approximately 2 min (Sharkey et al., 2007).

It is not possible to directly measure the movement of CO_2_ across the mesophyll. A number of methods have been developed that indirectly gauge *g*_m_ through calculation of *C*_i_ and the concentration of CO_2_ within the chloroplast envelope (*C*_c_) (Haworth et al., 2018). These methodologies all have inherent strengths and weaknesses (for a review of the methods of *g*_m_ calculation see Pons et al., 2009), leading to a preference in many studies to utilise at least two approaches to measure *g*_m_ (e.g. Haworth et al., 2022). In the present study, we utilised the variable J (Harley et al., 1992), curve fitting (Ethier & Livingston, 2004) and anatomy‐derived methods (Tomás et al., 2013, see the section ‘Analysis of leaf anatomical and structural traits’) to determine *g*_m_.

Mesophyll conductance was calculated using the variable J method (Harley et al., 1992; Loreto et al., 1992) as follows:

(2) $$g_{m}=\frac{A}{C_{i}-\frac{\Gamma^{*}\cdot \left[\mathrm{ETR}+8\cdot \left(A+R_{d}\right)\right]}{\mathrm{ETR}-4\cdot \left(A+R_{d}\right)}},$$

where the CO_2_ compensation point in the absence of respiration in the light (Γ*) was determined with the RubisCO specificity factor reported for deciduous woody species by Galmes et al. (2005), that is 96.6 at 25°C. The respiration rate in the light (*R*_d_) was assumed to be half of the dark respiration rate (*R*_n_) as suggested in previous studies (Centritto et al., 2009; Niinemets et al., 2005). The rate of *R*_n_ at 25°C was obtained from nighttime measurements (20:00 to 21:00 h) where the LED light source of the LI‐6800 was switched off (i.e. PPFD = 0 μmol m^−2^ sec^−1^). At the same time, nighttime stomatal conductance (*g*_night_) was also obtained in this measurement. The CO_2_ concentration inside the chloroplast envelope (*C*_c_) was calculated using the value of *g*_m_ as *C*_c_ = *C*_i_ – *A*_sat_/*g*_m_. For the calculation of the rate of electron transport (ETR) and photorespiration (*R*_PR_), see Protocol S1.

Total CO_2_ conductance (*g*_tot_) can be calculated as (Haworth et al., 2018):

(3) $$g_{\mathrm{tot}}=\left[\left(g_{s}/1.6\right)\cdot g_{m}\right]/\left[\left(g_{s}/1.6\right)+g_{m}\right].$$

The curve‐fitting method proposed by Ethier and Livingston (2004) following Farquhar et al. (1980) was applied to calculate the carboxylation capacity of RubisCO (*V*_cmax_), the maximum rate of electron transport required for ribulose‐1,5‐bisphosphate (RuBP) regeneration (*J*_max_) and *g*_m_ values using the *A*/*C*_i_ curve data.

According to Grassi and Magnani (2005), the relative limitations in *A*_sat_ (stomatal limitation, *l*_s_; mesophyll conductance limitation, *l*_m_; biochemical limitation, *l*_b_) were determined as follows:

(4) $$l_{s}=\frac{g_{\mathrm{tot}}/g_{s{\mathrm{CO}}_{2}}\cdot \delta A_{\mathrm{sat}}/\delta C_{c}}{g_{\mathrm{tot}}+\delta A_{\mathrm{sat}}/\delta C_{c}},$$

(5) $$l_{m}=\frac{g_{\mathrm{tot}}/g_{m}\cdot \delta A_{\mathrm{sat}}/\delta C_{c}}{g_{\mathrm{tot}}+\delta A_{\mathrm{sat}}/\delta C_{c}},$$

(6) $$l_{b}=\frac{g_{\mathrm{tot}}}{g_{\mathrm{tot}}+\delta A_{\mathrm{sat}}/\delta C_{c}},$$

where $g_{s{\mathrm{CO}}_{2}}$ is stomatal conductance for CO_2_ (=*g*_s_/1.6), *δA*_sat_/*δC*_c_ is an initial slope of *A*/*C*_c_ curves (*C*_c_: over a range of 0–150 μmol mol^−1^).

### Measurement of leaf abscisic acid, chlorophyll, leaf mass per area, leaf water and nitrogen content

After measuring the gas exchange rate, the leaf was cut into two equal halves with one half of the leaf used to determine abscisic acid (ABA) concentration. The leaf samples were immediately placed in liquid N and stored in a freezer at −80°C until the analysis. After extracting 0.1 g of leaf samples in 1 ml of distilled water, the resulting supernatant was further diluted 10 times according to the manufacturing protocol. The quantification of ABA was performed at 415 nm with a fluorescence/absorbance microplate reader (Victor3 1420 Multilabel Counter; Perkin Elmer, Waltham, MA, USA; Pisuttu et al., 2023) using the Phytodetek® Immunoassay Kit for ABA (Agdia, Elkhart, IN, USA).

Total chlorophyll content (Tot_Chl_) was quantified by Ultra‐High Performance Liquid Chromatography (UHPLC) using a Dionex UltiMate 3000 system (Thermo Scientific, Waltham, MA, USA). Chlorophylls were detected at 445 nm with a Dionex UVD 170 U UV–Vis detector (Thermo Scientific).

From the other half of the measured leaves, three leaf discs (1 cm diameter) per leaf were collected avoiding the main rib to determine leaf mass per unit area (LMA), leaf water content (LWC), and nitrogen (N) content. Leaf discs were dried in an oven at 70°C until their weight remained constant. Fresh and dry weights (FW and DW, respectively) were measured by a balance (Mod. BP110S; Sartorius, Goettingen, Germany). Leaf mass per area was calculated as the leaf DW:area ratio. Leaf water content was calculated as (FW – DW)/FW × 100. Leaf N content per unit mass (N_mass_) was determined by gas chromatography (NA1500 Analyser; Carlo Erba Instruments, Rodano, Italy). The N concentration per unit leaf area (N_area_) was then determined using N_mass_ and LMA. Photosynthetic N use efficiency (PNUE) was calculated as *A*_sat_/N_area_. In addition, mean LMA per treatment for each species was used to estimate total leaf area as leaf biomass/LMA using published leaf biomass data for the target poplar clones (Hoshika et al., 2024).

### Analysis of leaf anatomical and structural traits

Light and electron microscopies were applied for the anatomical analysis (Oguchi et al., 2003; Tosens et al., 2012). For the microscopic analysis of leaf structure, further leaf discs (0.8 cm diameter, three discs per leaf) were taken from intercostal and middle sections of leaves after the leaf gas exchange measurements. Discs were fixed in EM‐grade 2.5% glutaraldehyde solution which was buffered at pH 7.0 and with a 0.067 molar Soerensen phosphate buffer and stored at 4°C until further processing. Sample dehydration was performed using 2‐methoxyethanol (three changes), ethanol, *n*‐propanol, and *n*‐butanol and embedded in historesin. For light microscopy, 0.5‐μm semi‐thin cuttings were sectioned using an ultramicrotome Ultracut S (Reichert‐Yung, Wien, Austria). The cuttings were stained using Toluidine blue (Feder & O'Brien, 1968) and mounted in semi‐permanent slides using glycerin 50%. Observations using 20× to 100× objectives were performed with a system composed of a Nikon Eclipse E600 microscope, a Nikon Digital SIGHT DS‐SMc camera and NIS‐Elements F3.0 microscope camera image processing software (Nikon Corp, Tokyo, Japan). The following leaf structural parameters were investigated with ImageJ software (Schneider et al., 2012). Stomatal density on the upper (SD_upp_) and lower (SD_low_) leaf surfaces was assessed on paradermal view. Leaf thickness (*t*_leaf_), mesophyll thickness (*T*_mes_) and the fraction of intercellular air space (*F*_ias_) were measured from leaf transverse sections. Leaf density (*D*_leaf_) was calculated as LMA/*t*_leaf_. The mesophyll surface area facing the intercellular space per unit leaf area (*S*_mes_/*S*) and chloroplast surface area facing the intercellular space per unit leaf area (*S*_c_/*S*) were calculated considering a curvature correction factor (Thain, 1983) depending on the shape of the cell (palisade parenchyma tissue: 1.50 ± 0.03; spongy parenchyma tissue: 1.33 ± 0.08 as mean ± SE):

(7) $$S_{\mathrm{mes}}/S=\frac{l_{\mathrm{mes}}}{w}\times F,$$

(8) $$S_{c}/S=\frac{l_{c}}{l_{\mathrm{mes}}}\times \frac{l_{\mathrm{mes}}}{w}\times F,$$

where *l*_mes_ is the total length of mesophyll cells and *l*_c_ is the total length of chloroplasts facing the intercellular space, and *w* is the width of the measured section. In addition, the proportion of chloroplast‐covered area on the mesophyll surface area (*S*_c_/*S*_mes_) was calculated as [*S*_c_/*S*]/[*S*_mes_/*S*] according to Oguchi et al. (2003). For transmission electron microscopy (TEM), the samples were subjected to post‐fixation with 1% aqueous osmium tetroxide (OsO_4_) overnight. This was followed by washing in distilled water and dehydration in an ascending ethanol series for 1 h at each step, and the absolute ethanol series was repeated three times. The material was infiltrated and polymerized in LR White ‘hard grade’ resin (EMS®) according to the manufacturer's instructions. The ultrafine sections were prepared with a Leica ultramicrotome using a diamond knife and contrasted with uranyl acetate (Bozzola & Russel, 1998) and lead citrate (Hanaich et al., 1986). The sections were examined under a Tecnai G2 Spirit BioTWIN (FEI Company, Eindhoven, Netherlands) transmission electron microscope at 80 kV. According to the TEM image, cell wall thickness (*T*_cw_), chloroplast length and width (*L*_chl_ and *W*_chl_) were evaluated. All parameters were determined from the average of six measurements on each leaf.

According to the anatomical analysis, we estimated *g*_m_ following the method of Tomás et al. (2013) as:

(9) $$g_{m}=\frac{1}{\frac{1}{g_{\mathrm{ias}}}+\frac{\mathrm{RT}}{H\cdot g_{\mathrm{liq}}}},$$

where *g*_ias_ is the gas phase conductance and *g*_liq_ is the liquid phase conductance to CO_2_ diffusion inside the leaf, *R* is the gas constant (Pa m^3^ K^−1^), *T* is the temperature (K) and *H* is the Henry's law constant (m^3^ mol^−1^ K^−1^). *g*_ias_ is defined as:

(10) $$g_{\mathrm{ias}}=\frac{D_{a}\cdot F_{\mathrm{ias}}}{\Delta L_{\mathrm{ias}}\cdot t},$$

where *D*_a_ is the binary diffusion coefficient for CO_2_ in the air (1.51 × 10^−5^ m^2^ sec^−1^ at 25°C), Δ*L*_ias_ is the effective diffusion path length (=0.5 × *T*_mes_, Tomás et al., 2013), and *t* is the diffusion path tortuosity (1.57 m m^−1^, Syvertsen et al., 1995). According to Tomás et al. (2013), *g*_liq_ is given by:

(11) $$g_{\mathrm{liq}}=\frac{S_{c}}{S}\cdot \frac{1}{\left(r_{\mathrm{cw}}+r_{\mathrm{pl}}+r_{\mathrm{cel},1}\right)}+\left(\frac{S_{\mathrm{mes}}-S_{c}}{S}\right)\cdot \frac{1}{\left(r_{\mathrm{cw}}+r_{\mathrm{pl}}+r_{\mathrm{cel},2}\right)},$$

where *r*_sw_ is the cell wall resistance, *r*_pl_ is the plasmalemma resistance, *r*_cel,1_ is the partial liquid phase resistance for cell wall parts adjacent to chloroplasts, and *r*_cel,2_ is the partial liquid phase resistance for inter‐chloroplastial areas. For each liquid phase resistance, the following formula was applied (Tomás et al., 2013):

(12) $$g_{i}=\frac{1}{r_{i}}=\frac{r_{f,l}\cdot D_{w}\cdot p_{i}}{\Delta L_{i}},$$

where *r*_f,l_ is a dimensionless factor, which was set to 1.0 for the cell wall and 0.3 for the cytosol and stroma (Niinemets & Reichstein, 2003); *D*_w_ is the diffusion coefficient in the aqueous phase for CO_2_ (1.79 × 10^−9^ m^2^ sec^−1^ at 25°C); *p*_i_ is the effective porosity, which was set to 1.0 for the cell cytosol and stroma (Nobel, 2005). For the cell wall, a least‐squares iterative approach was applied to estimate *p*_i_ to achieve the optimal alignment between the measured and modeled *g*_m_, assuming that the *p*_i_ for the cell wall ranged from 0.028 to 0.3 (Tomás et al., 2013). Δ*L*_i_ is the diffusion path length of the corresponding component, which was estimated from the TEM images (Tomás et al., 2013).

### Data analysis

All statistical analyses were performed in R 4.1.2 (R Core Team, 2021). The effects of O_3_ on the number of attached, shed and total emerged leaves and leaf longevity were examined by analysis of variance (anova). Data were checked for normal distribution (Kolmogorov–Smirnov *D* test) and homogeneity of variance (Levene's test).

Spearman correlation analyses were made for all combinations of physiological and anatomical parameters. Also, linear regression analyses were performed to examine the relationships among physiological and anatomical parameters and leaf age. When the regressions were statistically significant (*p* ≤ 0.05) for at least two O_3_ treatments, an analysis of covariance (ancova) was applied to investigate the statistical difference between regression lines.

Ozone may affect physiological and morphological traits through multiple processes, including both direct and indirect effects. In addition, there is often a collinearity between the variables. To describe the complex structure of plant responses, a structural equation modelling (SEM) framework (Fan et al., 2016), which is a combined factorial and regression analytical method, was used to test the interactive effects of O_3_ and leaf age on *g*_m_ to investigate causal relationships. We assumed two latent variables, that is leaf structure (characterised by LMA, *t*_leaf_, *D*_leaf_, *T*_mes_, *F*_ias_, *S*_mes_/*S*, *S*_c_/*S*, *S*_c_/*S*_mes_, *SD*_upp_, *SD*_low_, *T*_cw_, *L*_chl_ and *W*_chl_) and leaf physiology (characterised by N_area_, ABA, Tot_Chl_, LWC). We used the R package ‘*lavaan*’ to develop an SEM and to identify potential causal relationships, assuming the following processes: (i) the interaction of O_3_ and leaf age affected leaf structural and/or leaf physiological traits, and (ii) leaf physiological and structural parameters affected *g*_m_. For leaf physiological and structural parameters, we have chosen the variables significantly correlated with *g*_m_ for both poplar clones, according to the above‐mentioned Spearman analysis. We then tested all of the possible combinations of cause‐effect relationships to select the observed variables for the final model to explain the entire structure according to the χ^2^ test, CFI (Comparative Fit Index) and BIC (Bayesian information criterion) (Fan et al., 2016). To sufficiently consider the variables, at least two observed variables for leaf structure or physiology and at least five observed variables in total were assessed. All target variables were normalised by *Z*‐transformation. For all statistical analyses, we used the values of *g*_m_ calculated from the variable J method. All statistical results were considered significant at *P* ≤ 0.05. R codes for our SEM analysis are provided in Protocol S2.

## AUTHOR CONTRIBUTIONS

Conceptualization, YH and EPa; data curation, YH, CP, LC, EPe, JLSM and BBM; formal analysis, YH and BBM; funding acquisition, YH, EPa, RVR and JLSM; investigation, YH, LC, CP, MH, EPe, CN, RVR, JLSM and BBM; writing‐original draft, YH; writing‐review and editing, EPa, CP, LC, MH, EPe, CN, RVR, JLSM and BBM.

## CONFLICT OF INTEREST

The authors declare that they have no conflicts of interest.

## Supporting information


**Appendix S1.** **Protocol S1.** Calculation of ETR and *R*_PR_.
**Protocol S2.** R codes for Structural Equation Model (SEM).
**Figure S1.** Changes in leaf length with time for two poplar clones.
**Figure S2.** Relationship between *V*_cmax_, *J*_max_ or *R*_PR_ and leaf age.
**Figure S3.** Relationship between *g*_night_, *R*_n_ or φPSII and leaf age.
**Figure S4.** Relationships between *A*_sat_ and diffusive parameters (*g*_s_, *g*_m_ and *g*_tot_).
**Figure S5.** Relationship between N_area_, Tot_Chl_, Ln[ABA] and leaf age.
**Figure S6.** Relationship between PNUE, *V*_cmax_/N_area_ and leaf age.
**Figure S7.** Relationship between LMA, LWC or *D*_leaf_ and leaf age.
**Figure S8.** Relationship between *t*_leaf_, *T*_mes_ or *F*_ias_ and leaf age.
**Figure S9.** Relationship between *S*_mes_/*S*, *S*_c_/*S* or *S*_c_/*S*_mes_ and leaf age.
**Figure S10.** Relationship between *T*_cw_, *L*_chl_ or *W*_chl_ and leaf age.
**Figure S11.** Relationships between stomatal density (SD_upp_ or SD_low_) and leaf age.
**Figure S12.** Relationships between *g*_m_ and *S*_c_/*S*, *T*_cw_, or Ln[ABA].
**Figure S13.** Leaf cross‐sections of young and old leaves by light microscope for two poplar clones grown with different O_3_ levels.
**Table S1.** Total leaf area at the end of the experiment for two poplar clones grown with different O_3_ levels.
**Table S2.** Candidate structural equation models (SEMs) to explain the interactive effects of O_3_ and leaf age on *g*_m_ for two poplar clones grown with different O_3_ levels.
**Table S3.** Leaf anatomical parameters (*t*_leaf_, *D*_leaf_, SD_upp_, SD_low_) for two poplar clones.
**Table S4.** Leaf anatomical parameters (*t*_mes_, *F*_ias_, *S*_mes_/*S*, *S*_c_/*S*, *S*_c_/*S*_mes_, *T*_cw_, *L*_chl_, *W*_chl_) for two poplar clones.

## Data Availability

All relevant data can be found within the manuscript and its supporting materials.
