# Supplementary material for: Leaf phenology determines the response of poplar genotypes to O3 through mesophyll conductance
Source: Plant J. 2025 Mar 7;121(5):e70091. doi: 10.1111/tpj.70091 (PMC11887005; doi:10.1111/tpj.70091)
Supplement: Supplementary file 1 — Appendix S1. Protocol S1. Calculation of ETR and R PR. Protocol S2. R codes for Structural Equation Model (SEM). Figure S1. Changes in leaf length with time for two poplar clones. Figure S2. Relationship between V cmax, J max or R PR and leaf age. Figure S3. Relationship between g night, R n or φPSII and leaf age. Figure S4. Relationships between A sat and diffusive parameters (g s, g m and g tot). Figure S5. Relationship between Narea, TotChl, Ln[ABA] and leaf age. Figure S6. Relationship between PNUE, V cmax/Narea and leaf age. Figure S7. Relationship between LMA, LWC or D leaf and leaf age. Figure S8. Relationship between t leaf, T mes or F ias and leaf age. Figure S9. Relationship between S mes/S, S c/S or S c/S mes and leaf age. Figure S10. Relationship between T cw, L chl or W chl and leaf age. Figure S11. Relationships between stomatal density (SDupp or SDlow) and leaf age. Figure S12. Relationships between g m and S c/S, T cw, or Ln[ABA]. Figure S13. Leaf cross‐sections of young and old leaves by light microscope for two poplar clones grown with different O3 levels. Table S1. Total leaf area at the end of the experiment for two poplar clones grown with different O3 levels. Table S2. Candidate structural equation models (SEMs) to explain the interactive effects of O3 and leaf age on g m for two poplar clones grown with different O3 levels. Table S3. Leaf anatomical parameters (t leaf, D leaf, SDupp, SDlow) for two poplar clones. Table S4. Leaf anatomical parameters (t mes, F ias, S mes/S, S c/S, S c/S mes, T cw, L chl, W chl) for two poplar clones. [file TPJ-121-0-s001.docx]

**Leaf phenology determines the response of poplar genotypes to O_3_ through mesophyll conductance**

Yasutomo Hoshika^1,2*^, Elena Paoletti^1,2^, Claudia Pisuttu^3^, Lorenzo Cotrozzi^3,4^, Matthew Haworth^5^, Elisa Pellegrini^3,4^ Cristina Nali^3,4^, Rafael Vasconcelos Ribeiro^6^, Juliana Lischka Sampaio Mayer^6^ and Barbara Baesso Moura^1,2^

*1. Institute of Research on Terrestrial Ecosystems (IRET), National Research Council of Italy (CNR), Via Madonna del Piano, I-50019 Sesto Fiorentino, Italy*

*2. NBFC, National Biodiversity Future Center, Palermo 90133, Italy*

*3. Department of Agriculture, Food and Environment, University of Pisa, Via del Borghetto, 80, 56124, Pisa, Italy*

*4. CIRSEC, Centre for Climate Change Impact, University of Pisa, Via del Borghetto 80, 56124 Pisa, Italy*

*5. Institute for the Sustainable Plant Protection (IPSP), National Research Council of Italy (CNR), Via Madonna del Piano, I-50019 Sesto Fiorentino, Italy*

*6. Department of Plant Biology, Institute of Biology, State University of Campinas (UNICAMP), Campinas SP, Brazil*

*Corresponding author: Yasutomo Hoshika, Tel: +39-055-522-5949, Fax: +39-055-522-5920 E-mail: [yasutomo.hoshika@cnr.it](mailto:yasutomo.hoshika@cnr.it)

**Supplementary Protocol S1: Calculation of ETR and *R*_PR_**

The rate of electron transport (ETR) was estimated from chlorophyll fluorescence using a single saturating pulse of 8000 μmol m^-2^ s^-1^ and duration of 1 second (Genty et al., 1989):

$ETR=PPFD\cdot\Phi PSII\cdot\alpha\cdot\beta$ (S1)

where α is the leaf absorbance, and *β* is the partitioning factor between photosystems I and II (Laisk & Loreto, 1996). Standard values of α = 0.85 and *β* = 0.5 were used (Gilbert et al., 2012; Laisk & Loreto, 1996). ΦPSII is the actual quantum efficiency of photosystem II, which can be calculated as:

$\Phi PSII=\frac{F_{m}^{'}-F_{s}}{F_{m}^{'}}$ (S2)

where *F*’_m_ is the maximal fluorescence and *F*_s_ is the steady state fluorescence under light adapted conditions (Genty et al. 1989). Photorespiration (*R*_PR_) was calculated as (Sharkey, 1988):

$R_{PR}=\frac{A+R_{d}}{\frac{C_{c}}{\Gamma^{*}}-1}$ (S3)

**Supplementary Protocol S2: R codes for Structural Equation Model (SEM).**

**a) SEM of mesophyll conductance for I-214 poplar**

dblink<-"./input/"

library("lavaan")

library(semPlot)

library(tidyverse)

library(lubridate)

library(dplyr)

library(psych)

library(car)

library(MuMIn)

test<-read.table(paste(dblink,"Poplar_2020_i214_SEM.txt", sep=""),sep="\t", stringsAsFactors = F, header = T)

O3xAge<-as.numeric(test$O3xAge)

Gm<-as.numeric(test$gm)

LMA<-as.numeric(test$LMA)

Narea<-as.numeric(test$Narea)

LWC<-as.numeric(test$LWC)

Chl<-as.numeric(test$TotChl)

tleaf<-as.numeric(test$tleaf)

Dleaf<-as.numeric(test$Dleaf)

Tmes<-as.numeric(test$Tmes)

Fias<-as.numeric(test$Fias)

SmesS<-as.numeric(test$Smes_S)

ScS<-as.numeric(test$Sc_S)

ScSmes<-as.numeric(test$Sc_Smes)

ABA<-as.numeric(test$ABA)

StDupp<-as.numeric(test$SDupp)

StDlow<-as.numeric(test$SDlow)

Tcw<-as.numeric(test$Tcw)

Lchl<-as.numeric(test$Lchl)

Wchl<-as.numeric(test$Wchl)

dat<-cbind(O3xAge,LMA,Narea,LWC,Gm,tleaf,Dleaf,Tmes,Fias,SmesS,ScS,ScSmes,Chl,ABA,StDupp,StDlow,Tcw,Lchl,Wchl)

# Standardisation

z <- scale(dat)

zz <- data.frame(z)

names(zz)<-c("O3xAge","LMA","Narea","LWC","Gm","tleaf","Dleaf","Tmes","Fias","SmesS","ScS","ScSmes","Chl","ABA","StDupp","StDlow","Tcw","Lchl","Wchl")

#####STRUCTURAL EQUATION MODEL#####

model.test <- '

#Definition of "latent variables"

LF_str =~ ScS + ScSmes + Tcw

LF_phys =~ Narea + LWC

LF_str ~ O3xAge

LF_phys ~ O3xAge

Gm ~ LF_str + LF_phys

LF_str ~~ LF_phys

'

#####END#####

result.test <- sem(model.test ,data=zz)

summary(result.test , standardized=TRUE , fit.measures=TRUE , modindices=F)

fitMeasures(result.test , c("gfi", "agfi","rmsea","cfi","pvalue","bic","bic2","aic"))

# Path Diagram

semPaths(result.test, "std", style="lisrel", layout = "tree", mar=c(6,1,3,1), edge.label.cex=.8,

fade=F, theme = "gray", label.cex = 1, asize = 3.0, edge.width = 0.3,

optimizeLatRes = TRUE, edge.label.position = 0.3, nCharNodes=10)

**b) SEM of mesophyll conductance for Oxford poplar**

dblink<-"./input/"

library("lavaan")

library(semPlot)

library(tidyverse)

library(lubridate)

library(dplyr)

library(psych)

library(car)

library(MuMIn)

test<-read.table(paste(dblink,"Poplar_2020_Oxford_SEM.txt", sep=""),sep="\t", stringsAsFactors = F, header = T)

O3xAge<-as.numeric(test$O3xAge)

Gm<-as.numeric(test$gm)

LMA<-as.numeric(test$LMA)

Narea<-as.numeric(test$Narea)

LWC<-as.numeric(test$LWC)

Chl<-as.numeric(test$TotChl)

tleaf<-as.numeric(test$tleaf)

Dleaf<-as.numeric(test$Dleaf)

Tmes<-as.numeric(test$Tmes)

Fias<-as.numeric(test$Fias)

SmesS<-as.numeric(test$Smes_S)

ScS<-as.numeric(test$Sc_S)

ScSmes<-as.numeric(test$Sc_Smes)

ABA<-as.numeric(test$ABA)

StDupp<-as.numeric(test$SDupp)

StDlow<-as.numeric(test$SDlow)

Tcw<-as.numeric(test$Tcw)

Lchl<-as.numeric(test$Lchl)

Wchl<-as.numeric(test$Wchl)

dat<-cbind(O3xAge,LMA,Narea,LWC,Gm,tleaf,Dleaf,Tmes,Fias,SmesS,ScS,ScSmes,Chl,ABA,StDupp,StDlow,Tcw,Lchl,Wchl)

# Standardisation

z <- scale(dat)

zz <- data.frame(z)

names(zz)<-c("O3xAge","LMA","Narea","LWC","Gm","tleaf","Dleaf","Tmes","Fias","SmesS","ScS","ScSmes","Chl","ABA","StDupp","StDlow","Tcw","Lchl","Wchl")

#####STRUCTURAL EQUATION MODEL#####

model.test <- '

#Definition of "latent variables"

LF_str =~ ScS + ScSmes + Dleaf

LF_phys =~ ABA + Chl

LF_str ~ O3xAge

LF_phys ~ O3xAge

Gm ~ LF_str + LF_phys

LF_str ~~ LF_phys

'

#####END#####

result.test <- sem(model.test ,data=zz)

summary(result.test , standardized=TRUE , fit.measures=TRUE , modindices=F)

fitMeasures(result.test , c("gfi", "agfi","rmsea","cfi","pvalue","bic","bic2","aic"))

# Path Diagram

semPaths(result.test, "std", style="lisrel", layout = "tree", mar=c(6,1,3,1), edge.label.cex=.8,

fade=F, theme = "gray", label.cex = 1, asize = 3.0, edge.width = 0.3,

optimizeLatRes = TRUE, edge.label.position = 0.3, nCharNodes=10)

**Supplementary figures and tables**


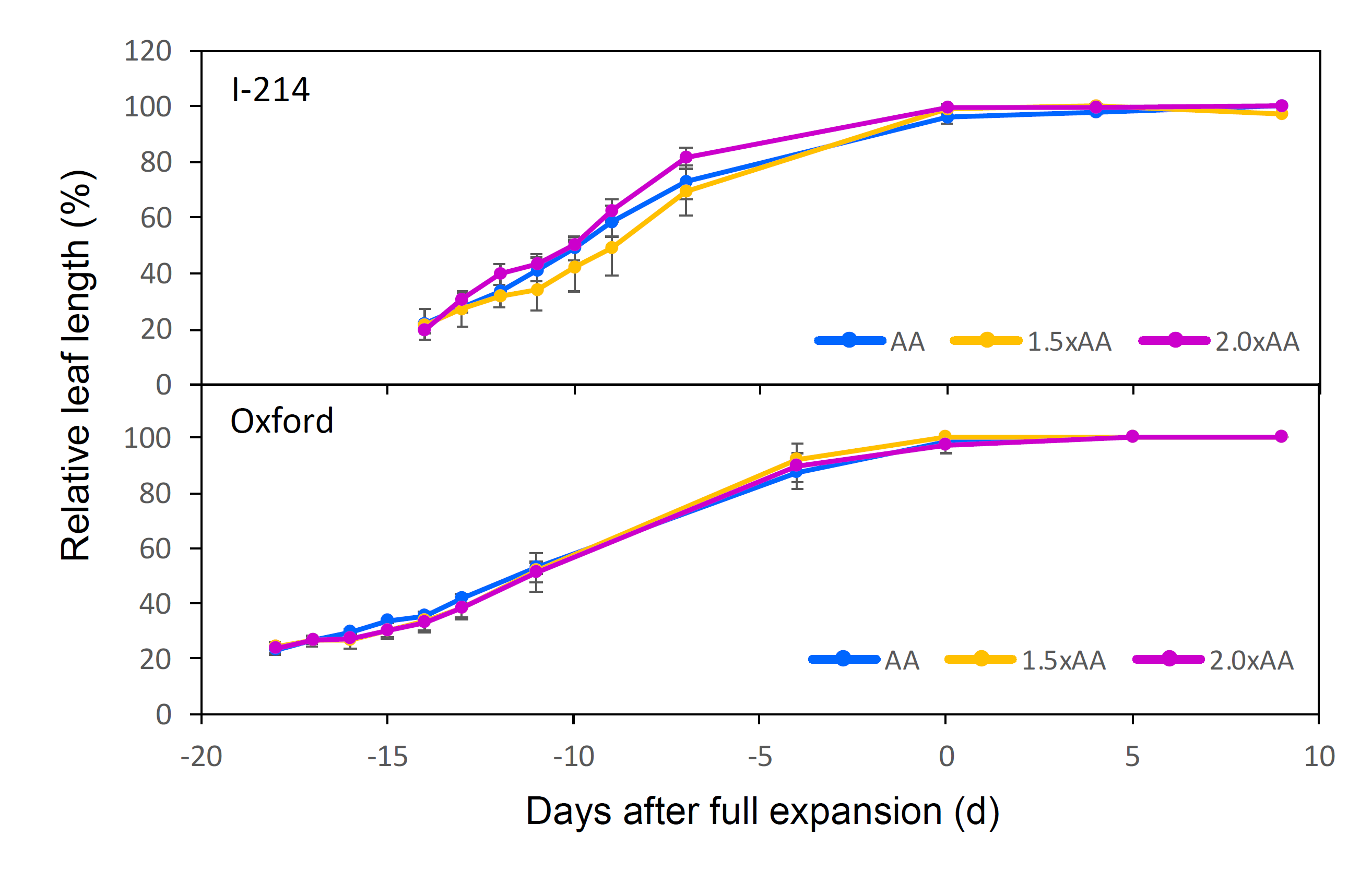


**Figure S1.** Changes in leaf length with time in I-214 and Oxford poplar clones grown at three O_3_ levels (AA, ambient O_3_ concentration, 1.5×AA, 2.0×AA). The symbols represent mean±S.E. (n = 3 plots). No significant difference among O_3_ treatments was observed by ANOVA.


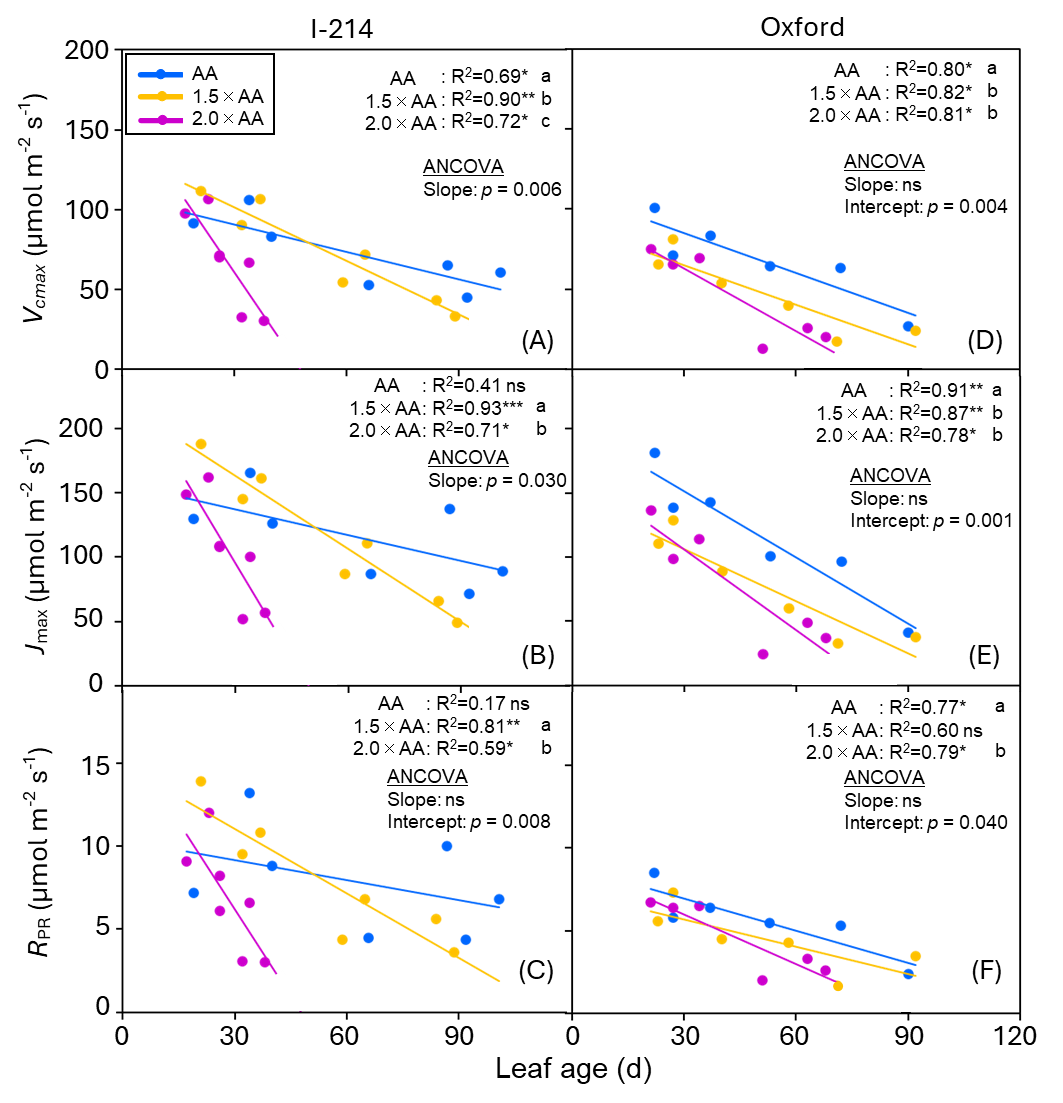


**Figure S2.** Relationships between the maximum rate of RubisCO carboxylation [*V*_cmax_] (A, D), the maximum rate of electron transport for RuBP regeneration [*J*_max_] (B, E) or photorespiration [*R*_PR_] (C, F) and leaf age in I-214 (A-C) and Oxford (D-F) poplar clones grown with three O_3_ levels (AA, ambient O_3_ concentration, 1.5×AA, 2.0×AA) (n = 3 plants). Linear regression analysis: *** *p* ≤ 0.001, ** *p* ≤ 0.01, * *p* ≤ 0.05, ns denotes not significant. When at least two regression lines were statistically significant, an ANCOVA was applied to examine a difference of the regressions between O_3_ treatments. Different letters denote significant differences of the regressions between O_3_ treatments (*p* ≤ 0.05).


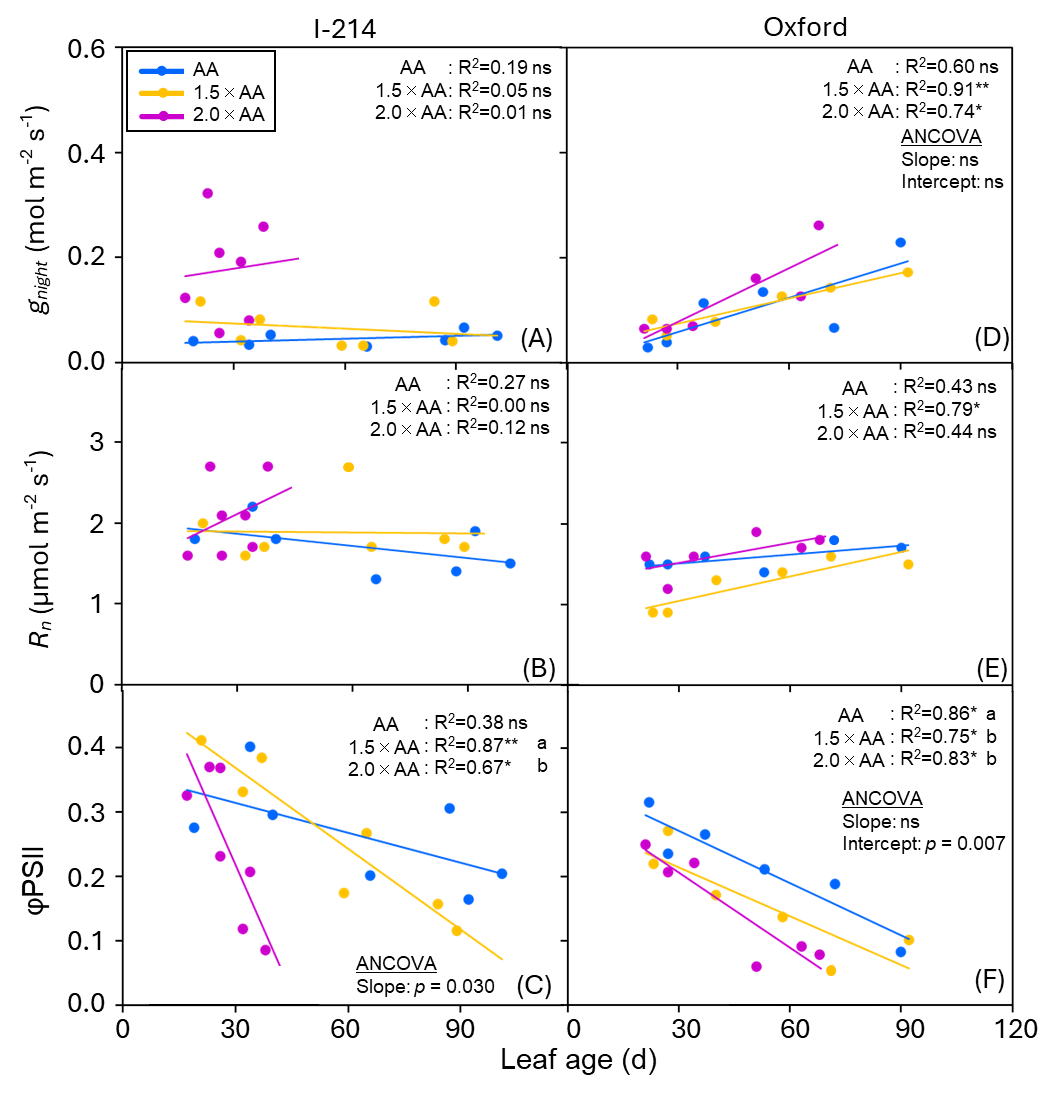


**Figure S3.** Relationships between nighttime stomatal conductance [*g*_night_] (A, D), dark respiration [*R*_n_] (B, E) or quantum yield of photosystem II photochemistry [φPSII] (C, F) or and leaf age in I-214 (A-C) and Oxford (D-F) poplar clones grown with three O_3_ levels (AA, ambient O_3_ concentration, 1.5×AA, 2.0×AA) (n = 3 plants). Linear regression analysis: ** *p* ≤ 0.01, * *p* ≤ 0.05, ns denotes not significant. When at least two regression lines were statistically significant, ANCOVA was applied to examine the difference of the regressions among O_3_ treatments. Different letters denote significant differences of the regression between O_3_ treatments (*p* ≤ 0.05).


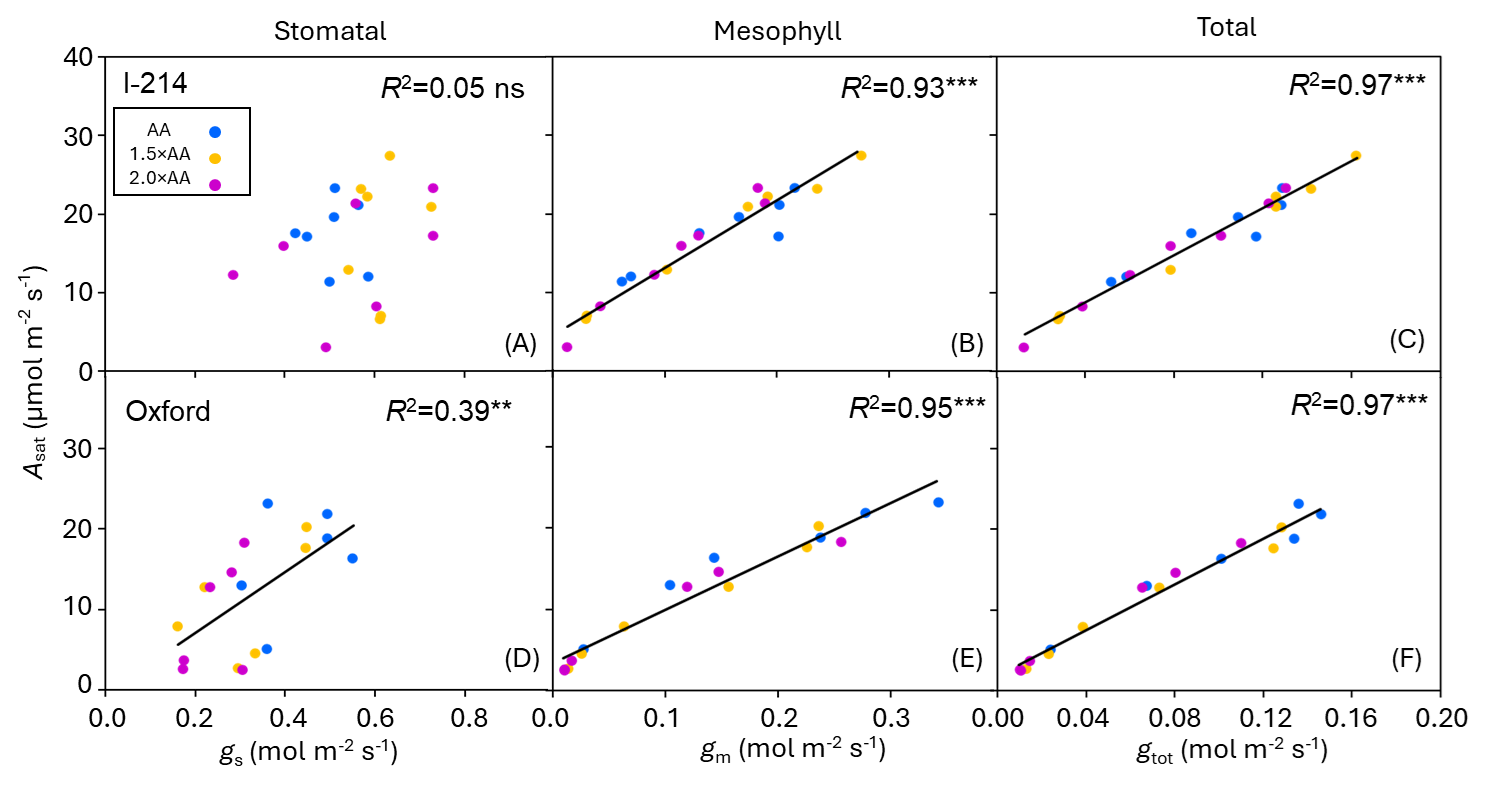


**Figure S4.** Relationships between (A, D) light-saturated net photosynthetic rate [*A*_sat_] and stomatal conductance [*g*_s_], (B, E) *A*_sat_ and mesophyll conductance [*g*_m_], (C, F) *A*_sat_ and total conductance to CO_2_ [*g*_tot_] in I-214 (A-C) and Oxford (D-F) poplar clones grown at three O_3_ levels (AA, ambient O_3_ concentration, 1.5×AA, 2.0×AA) (n = 3 plants). Linear regression analysis: *** *p* ≤ 0.001, ** *p* ≤ 0.01, ns denotes not significant.


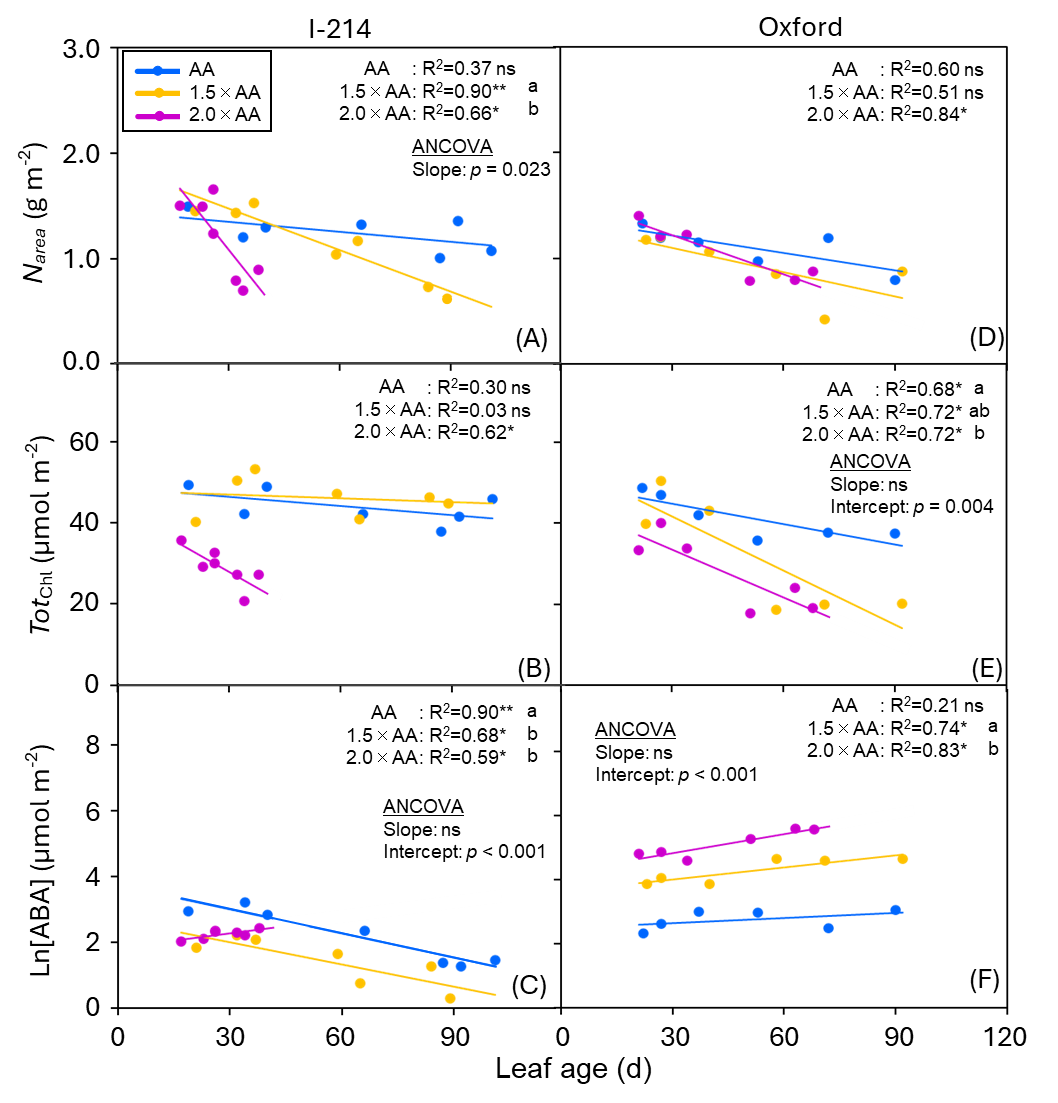


**Figure S5.** Relationships between leaf nitrogen content per unit leaf area [N_area_] (A, D), total chlorophyll content [*Tot*_Chl_] (B, E) or log-transformed foliar abscisic acid concentration [Ln(ABA)] (C, F), and leaf age in I-214 (A-C) and Oxford (D-F) poplar clones grown under three O_3_ levels [AA (ambient O_3_ concentration), 1.5×AA and 2.0×AA) (n = 3 plants). Linear regression analysis: **: *p* ≤ 0.01, *: *p* ≤ 0.05, ns denotes not significant. When at least two regression lines were statistically significant, ANCOVA was applied to examine the difference of the regressions among O_3_ treatments. Different letters denote significant differences of the regression among O_3_ treatments (*p* ≤ 0.05).

**
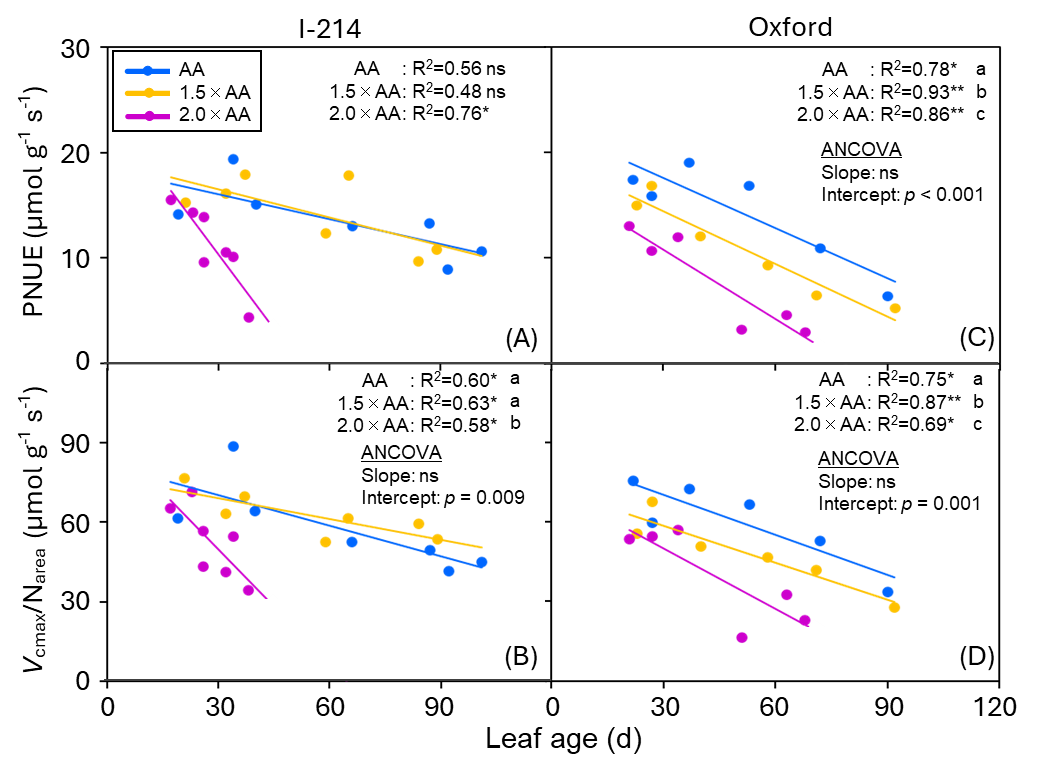
**

**Figure S6.** Relationships between photosynthetic nitrogen use efficiency [PNUE] (A, C) or the *V*_cmax_/N_area_ ratio (B, D) and leaf age in I-214 (A, B) and Oxford (C, D) poplar clones grown under three O_3_ levels [AA (ambient O_3_ concentration), 1.5×AA and 2.0×AA) (n = 3 plants). Linear regression analysis: **: *p* ≤ 0.01, *: *p* ≤ 0.05, ns denotes not significant. When at least two regression lines were statistically significant, ANCOVA was applied to examine the difference of the regressions among O_3_ treatments. Different letters denote significant differences of the regression among O_3_ treatments (*p* ≤ 0.05).


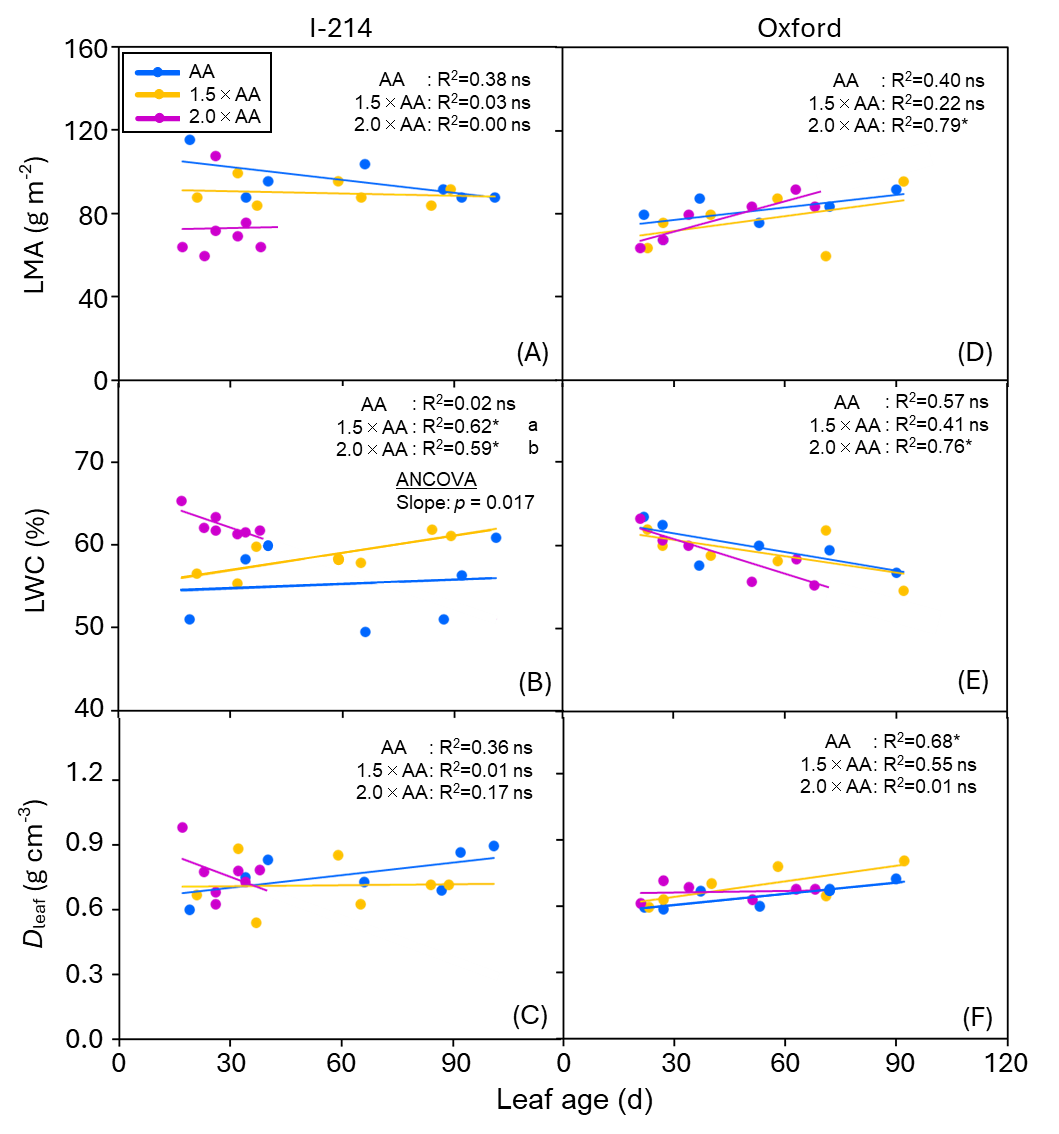


**Figure S7.** Relationships between leaf mass per area [LMA] (A, D), leaf water content [LWC] (B, E) or leaf density [*D*_leaf_] (C, F), and leaf age in I-214 (A-C) and Oxford (D-F) poplar clones grown under three O_3_ levels [AA (ambient O_3_ concentration), 1.5×AA and 2.0×AA) (n = 3 plants). Linear regression analysis: *: *p* ≤ 0.05, ns denotes not significant. When at least two regression lines were statistically significant, ANCOVA was applied to examine the difference of the regressions among O_3_ treatments. Different letters denote significant differences of the regression among O_3_ treatments (*p* ≤ 0.05).


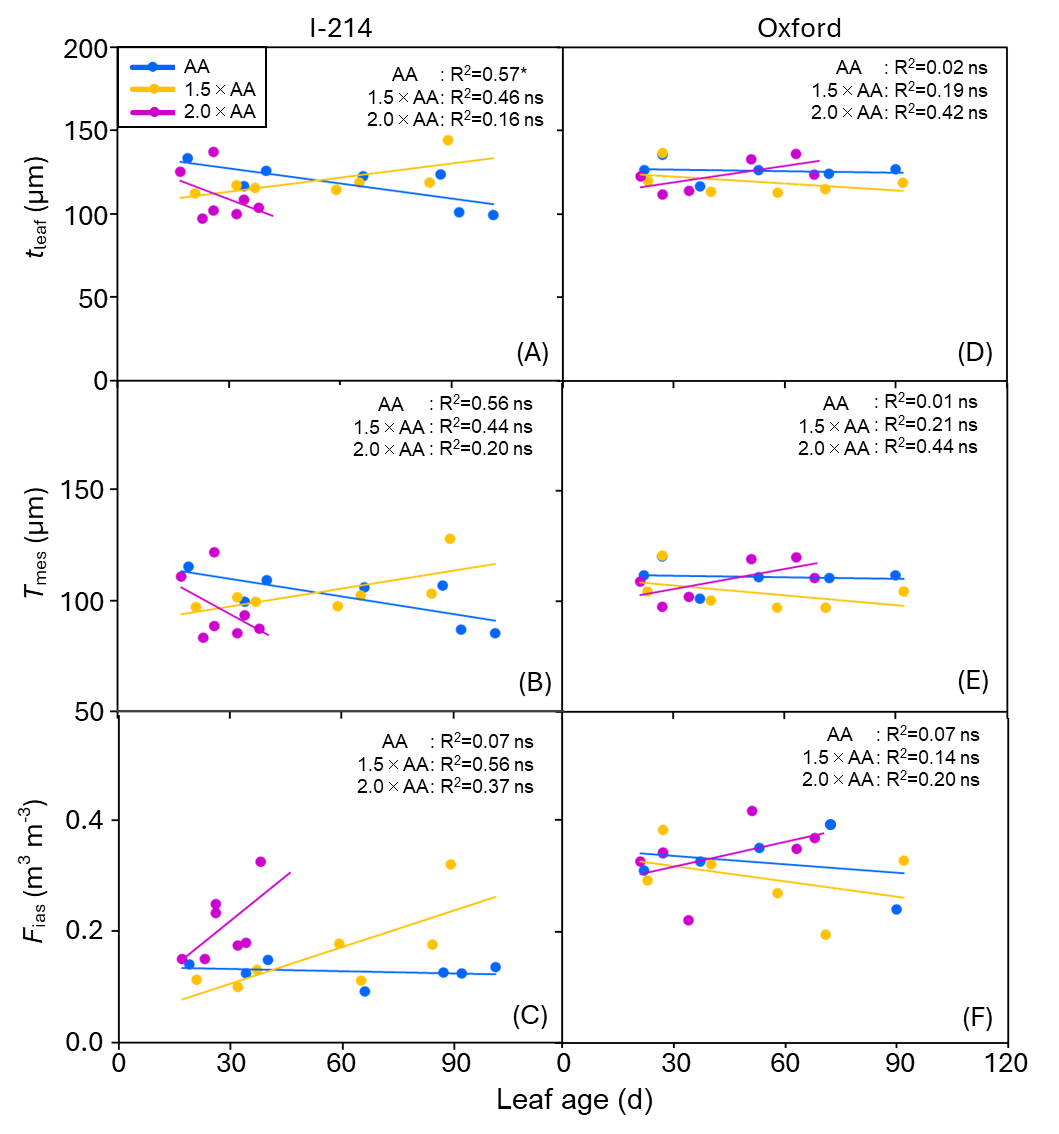


**Figure S8.** Relationships between leaf thickness [*t*_leaf_] (A, D), mesophyll thickness [*T*_mes_] (B, E) or intercellular air space [*F*_ias_] (C, F), and leaf age in I-214 (A-C) and Oxford (D-F) poplar clones grown under three O_3_ levels [AA (ambient O_3_ concentration), 1.5×AA and 2.0×AA) (n = 3 plants). Linear regression analysis: *: *p* ≤ 0.05, ns denotes not significant. When at least two regression lines were statistically significant, ANCOVA was applied to examine the difference of the regressions among O_3_ treatments. Different letters denote significant differences of the regression among O_3_ treatments (*p* ≤ 0.05).


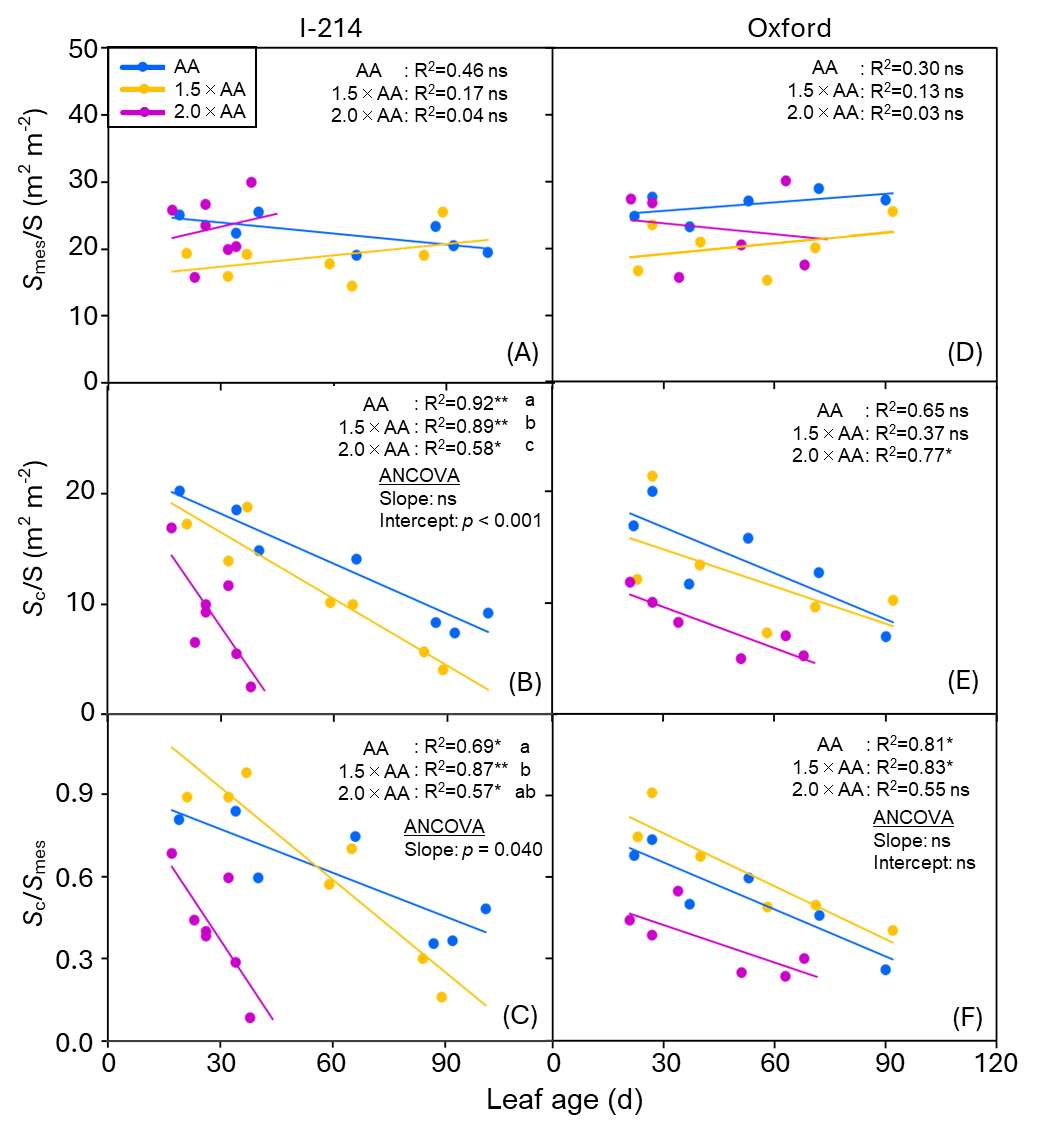


**Figure S9.** Relationships between mesophyll surface area and facing an intercellular space per unit leaf area [*S*_mes_/*S*] (A, D), chloroplast surface area facing an intercellular space per unit leaf area [*S*_c_/*S*] (B, E) and a proportion of chloroplast covered area on mesophyll cell surface [*S*_c_/*S*_mes_] (C, F) and leaf age in I-214 (A-C) and Oxford (D-F) poplar clones grown at three O_3_ levels (AA, ambient O_3_ concentration, 1.5×AA, 2.0×AA) (n = 3 plants). Linear regression analysis: ** *p* ≤ 0.01, * *p* ≤ 0.05, ns denotes not significant. When at least two regression lines were statistically significant, ANCOVA was applied to examine the difference of the regressions among O_3_ treatments. Different letters denote significant differences of the regression between O_3_ treatments (*p* ≤ 0.05).


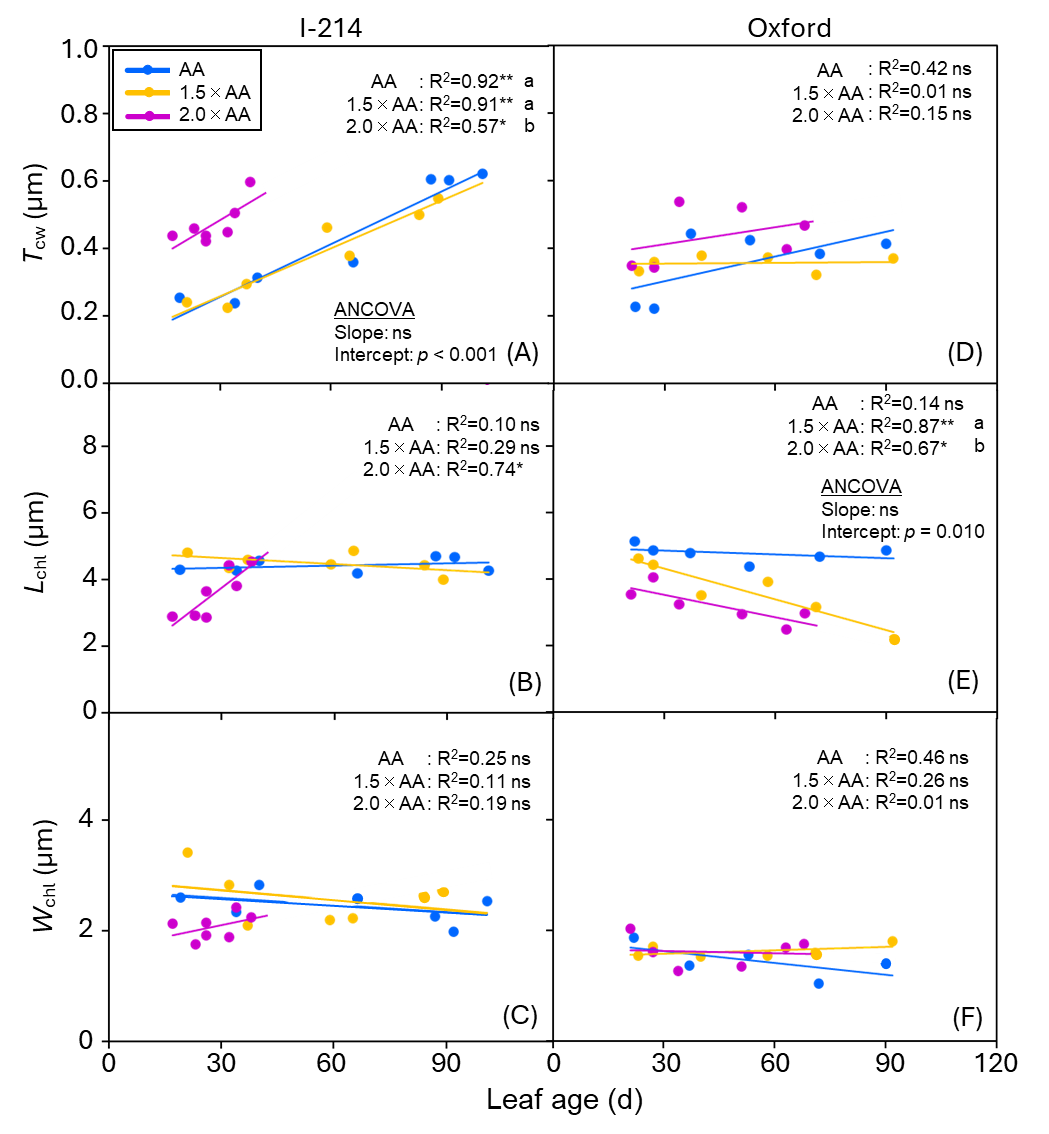


**Figure S10.** Relationships between cell wall thickness [*T*_cw_] (A, D), chloroplast length [*L*_chl_] (B, E) and chloroplast width [*W*_chl_] (C, F) and leaf age in I-214 (A-C) and Oxford (D-F) poplar clones grown at three O_3_ levels (AA, ambient O_3_ concentration, 1.5×AA, 2.0×AA) (n = 3 plants). Linear regression analysis: ** *p* ≤ 0.01, * *p* ≤ 0.05, ns denotes not significant. When at least two regression lines were statistically significant, ANCOVA was applied to examine the difference of the regressions among O_3_ treatments. Different letters denote significant differences of the regression between O_3_ treatments (*p* ≤ 0.05).


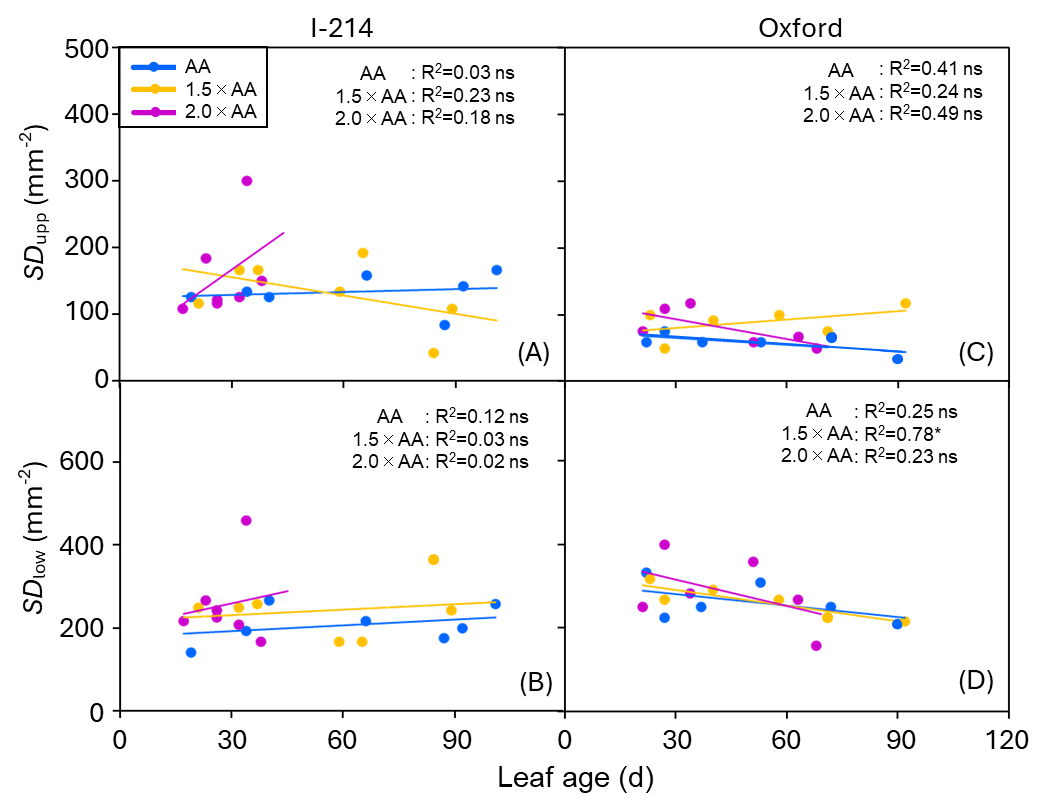


**Figure S11.** Relationships between stomatal density on upper leaf surface [*SD*_upp_] (A, C) or stomatal density on lower leaf surface [*SD*_low_] (B, D) and leaf age in I-214 (A, B) and Oxford (C, D) poplar clones grown at three O_3_ levels (AA, ambient O_3_ concentration, 1.5×AA, 2.0×AA) (n = 3 plants). Linear regression analysis: * *p* ≤ 0.05, ns denotes not significant.


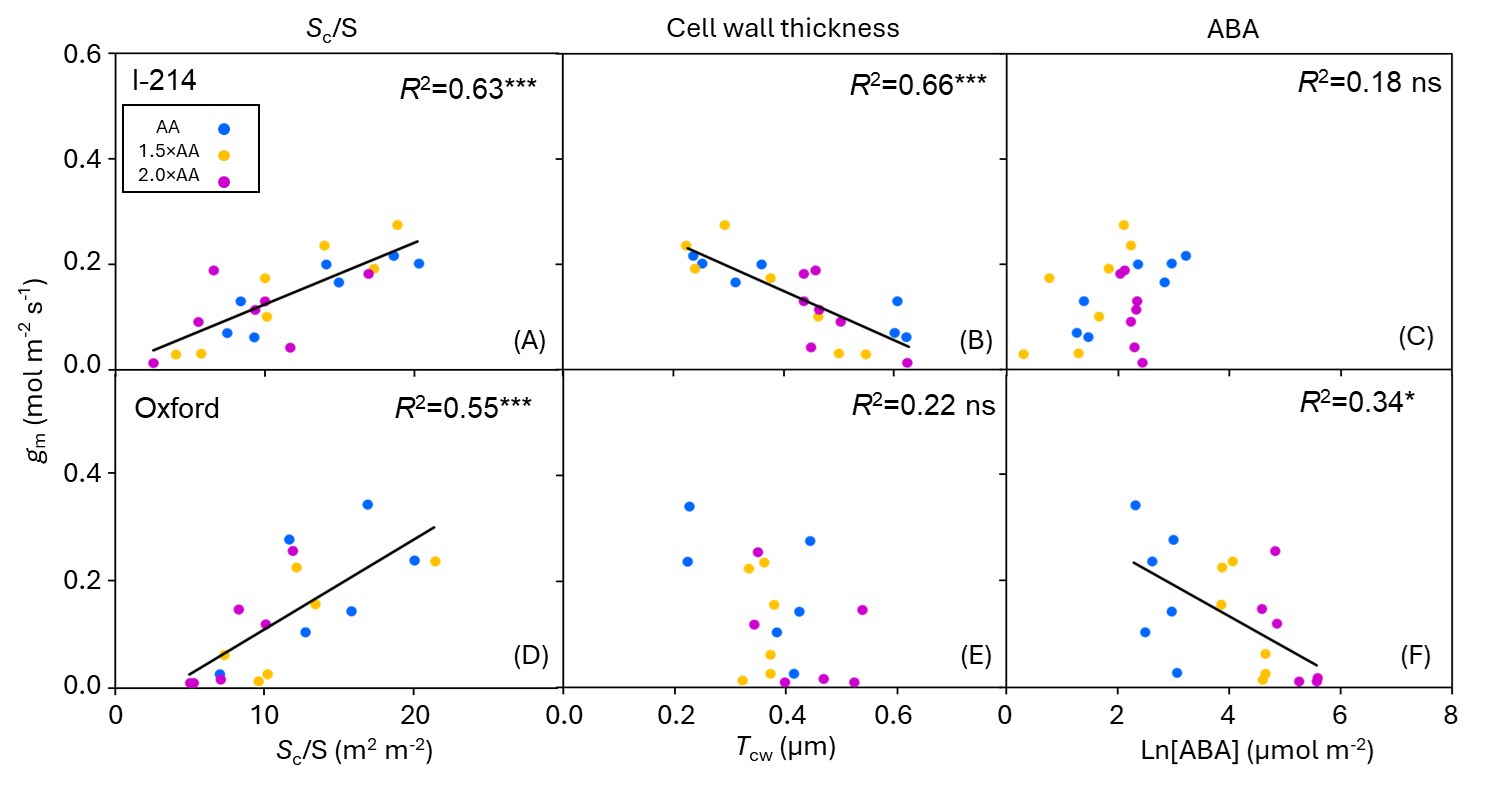


**Figure S12.** Relationships between mesophyll conductance [*g*_m_] and chloroplast surface area facing an intercellular space per unit leaf area [*S*_c_/*S*] (A, D), cell wall thickness [*T*_cw_] (B, E) or a log-transformed foliar abscisic acid [ABA] concentration (C, F) in I-214 (A-C) and Oxford (D-F) poplar clone grown with three O_3_ levels (AA, ambient O_3_ concentration, 1.5×AA, 2.0×AA) (n = 3 plants). Linear regression analysis: *** *p* < 0.001, * *p* < 0.05, ns denotes not significant.


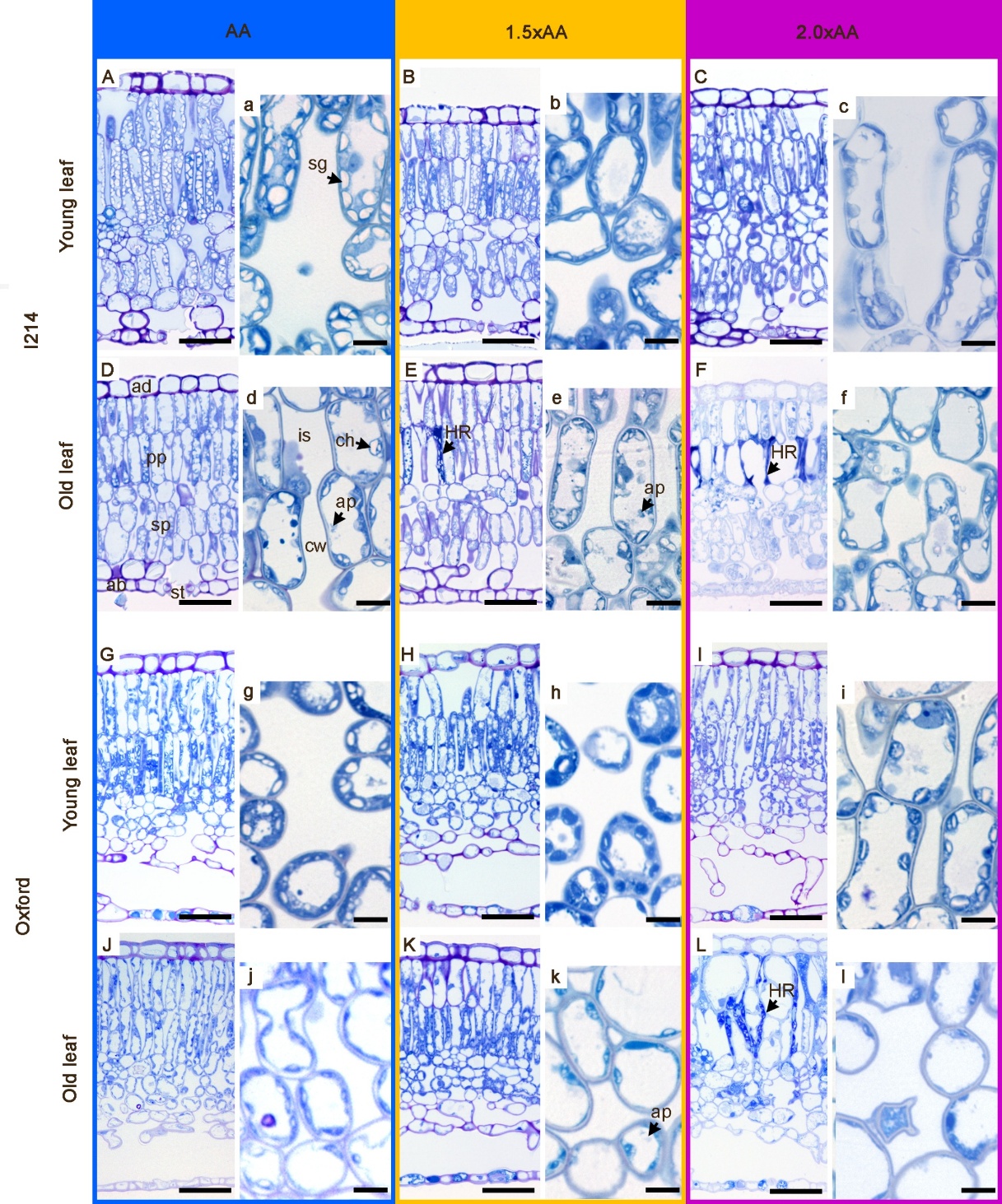


**Figure S13.** Leaf cross-sections of young and old leaves for I-214 (A-F) and Oxford (G-L) poplar clones grown at three O_3_ levels (AA, ambient O_3_ concentration, 1.5×AA, 2.0×AA). Leaf age of young leaves, I-214: 19 days in AA, 21 days in 1.5×AA and 17 days in 2.0×AA; Oxford: 22 days in AA, 23 days in 1.5×AA and 21 days in 2.0×AA. Leaf age of old leaves, I-214: 101 days in AA, 89 days in 1.5×AA and 38 days in 2.0×AA; Oxford: 90 days in AA, 92 days in 1.5×AA and 68 days in 2.0×AA. ad: adaxial surface; ab: abaxial surface; pp: palisade parenchyma; sp: spongy parenchyma; st: stomata; is: intercellular space; ch: chloroplast; cw: cell wall; HR: hypersensitive response. Bars in figures with capital letters = 50 µm; bars in figures with lowercase letters = 10 µm.

**Table S1.** Total leaf area at the end of the experiment (31 October) in I-214 and Oxford poplar clones grown at three O_3_ levels (AA, ambient O_3_ concentration, 1.5×AA, 2.0×AA). Data are mean ± SE (n = 3 plots). Asterisks show the significance of ANOVA: *** *p* ≤ 0.001, ** *p* ≤ 0.01, * *p* ≤ 0.05. Different letters show significant differences among treatments (*p* ≤ 0.05, Tukey test).

| Clone | O_3_ | Total leaf area |  |
| --- | --- | --- | --- |
|  |  | (cm^2^) |  |
| I-214 | AA | 1048 ± 24 a |  |
|  | 1.5×AA | 907 ± 89 ab |  |
|  | 2.0×AA | 486 ± 109 cd |  |
| Oxford | AA | 603 ± 48 bc |  |
|  | 1.5×AA | 501 ± 77 cd |  |
|  | 2.0×AA | 256 ± 63 d |  |
| ***ANOVA results*** | |  |  |
| O_3_ |  | *** |  |
| Clone |  | *** |  |
| O_3_ × Clone |  | ns |  |

**Table S2.** Candidate structural equation models (SEMs) to explain the interactive effects of O_3_ and leaf age on *g*_m_ considering causal relationships between leaf structural and physiological parameters in I-214 and Oxford poplar clones grown at three O_3_ levels (AA, ambient O_3_ concentration, 1.5×AA, 2.0×AA). All possible combinations of observed variables were used to select the best model according to χ^2^ test, CFI (Comparative Fit Index) and BIC (Bayesian information criterion).

|  |  |  |  |  | Combination of selected parameters | |  |
| --- | --- | --- | --- | --- | --- | --- | --- |
|  | Clone | Rank | Model performance | Criteria | Leaf structure | Leaf physiology |  |
|  | I-214 | 1 | *p*-value (χ^2^ test): 0.145 | *p* > 0.05 | *Sc/S, S*_c_/*S*_mes_, *T*_cw_ | N_area_, LWC |  |
|  |  |  | CFI: 0.961 | CFI > 0.9 |  |  |  |
|  |  |  | BIC: 269.474 |  |  |  |  |
|  |  | 2 | *p*-value (χ^2^ test): 0.097 | *p* > 0.05 | *Sc/S, S*_c_/*S*_mes_, *F*_ias_ | N_area_, LWC |  |
|  |  |  | CFI: 0.943 | CFI > 0.9 |  |  |  |
|  |  |  | BIC: 283.560 |  |  |  |  |
|  |  | 3 | *p*-value (χ^2^ test): 0.178 | *p* > 0.05 | *S*_c_/*S*, *T*_cw_, *F*_ias_ | N_area_, LWC |  |
|  |  |  | CFI: 0.955 | CFI > 0.9 |  |  |  |
|  |  |  | BIC: 302.414 |  |  |  |  |
|  | Oxford | 1 | *p*-value (χ^2^ test): 0.549 | *p* > 0.05 | *S*_c_/*S*, *S*_c_/*S*_mes_, *D*_leaf,_ | ABA, Tot_Chl_ |  |
|  |  |  | CFI: 1.000 | CFI > 0.9 |  |  |  |
|  |  |  | BIC: 228.391 |  |  |  |  |
|  |  | 2 | *p*-value (χ^2^ test): 0.569 | *p* > 0.05 | *S*_c_/*S*, *S*_c_/*S*_mes_, *D*_leaf,_ | ABA, LWC |  |
|  |  |  | CFI: 1.000 | CFI > 0.9 |  |  |  |
|  |  |  | BIC: 240.469 |  |  |  |  |
|  |  | 3 | *p*-value (χ^2^ test): 0.572 | *p* > 0.05 | *S*_c_/*S*, *S*_c_/*S*_mes_, *D*_leaf,_ | ABA, Tot_Chl_, LWC |  |
|  |  |  | CFI: 1.000 | CFI > 0.9 |  |  |  |
|  |  |  | BIC: 270.158 |  |  |  |  |

**Table S3.** Average (±S.E.) leaf thickness (*t*_leaf_, μm), density (*D*_leaf_, g cm^-3^), stomatal density on upper leaf surface (*SD*_upp_, mm^-2^) and stomatal density on lower leaf surface (*SD*_low_, mm^-2^) in I-214 and Oxford poplar clones grown at three O_3_ levels (AA, ambient O_3_ concentration, 1.5×AA, 2.0×AA). Leaf positions are denoted as “leaf order from the tip of the shoot/total number of attached leaves”.

| Clone | O_3_ | Leaf age | Leaf | *t*_leaf_ | *D*_leaf_ | *SD*_upp_ | *SD*_low_ |
| --- | --- | --- | --- | --- | --- | --- | --- |
|  |  |  | position | (μm) | (g cm^-3^) | (mm^-2^) | (mm^-2^) |
| I-214 | AA | 19 | 3/33 | 133.2 ± 2.3 | 0.60 ± 0.01 | 125 ± 22 | 142 ± 19 |
|  | AA | 34 | 7/33 | 116.5 ± 1.3 | 0.75 ± 0.01 | 133 ± 14 | 192 ± 40 |
|  | AA | 40 | 6/30 | 126.0 ± 0.5 | 0.83 ± 0.00 | 125 ± 28 | 267 ± 36 |
|  | AA | 66 | 12/33 | 122.4 ± 0.8 | 0.73 ± 0.01 | 158 ± 19 | 217 ± 30 |
|  | AA | 87 | 21/32 | 123.6 ± 1.3 | 0.69 ± 0.01 | 83 ± 17 | 175 ± 30 |
|  | AA | 92 | 27/34 | 100.8 ± 2.2 | 0.87 ± 0.02 | 142 ± 26 | 200 ± 33 |
|  | AA | 101 | 22/31 | 99.4 ± 2.0 | 0.90 ± 0.02 | 167 ± 19 | 258 ± 29 |
|  | 1.5×AA | 21 | 4/24 | 112.2 ± 1.5 | 0.67 ± 0.01 | 117 ± 32 | 250 ± 26 |
|  | 1.5×AA | 32 | 8/33 | 117.2 ± 1.0 | 0.88 ± 0.01 | 167 ± 26 | 250 ± 29 |
|  | 1.5×AA | 37 | 11/22 | 115.7 ± 0.9 | 0.54 ± 0.00 | 167 ± 26 | 258 ± 50 |
|  | 1.5×AA | 59 | 17/33 | 114.5 ± 1.7 | 0.85 ± 0.01 | 133 ± 26 | 167 ± 31 |
|  | 1.5×AA | 65 | 18/22 | 119.0 ± 0.4 | 0.63 ± 0.00 | 192 ± 40 | 167 ± 36 |
|  | 1.5×AA | 84 | 21/24 | 118.8 ± 1.4 | 0.72 ± 0.01 | 42 ± 15 | 367 ± 31 |
|  | 1.5×AA | 89 | 26/33 | 144.4 ± 2.4 | 0.72 ± 0.01 | 108 ± 15 | 242 ± 29 |
|  | 2.0×AA | 17 | 3/13 | 125.4 ± 1.4 | 0.98 ± 0.01 | 108 ± 23 | 217 ± 21 |
|  | 2.0×AA | 23 | 5/12 | 97.0 ± 0.7 | 0.78 ± 0.01 | 183 ± 17 | 267 ± 33 |
|  | 2.0×AA | 26 | 5/16 | 102.2 ± 0.8 | 0.68 ± 0.01 | 117 ± 27 | 242 ± 48 |
|  | 2.0×AA | 26 | 8/13 | 137.2 ± 1.1 | 0.63 ± 0.01 | 121 ± 20 | 225 ± 39 |
|  | 2.0×AA | 32 | 13/13 | 99.7 ± 1.1 | 0.78 ± 0.01 | 125 ± 13 | 208 ± 29 |
|  | 2.0×AA | 34 | 11/16 | 108.4 ± 0.9 | 0.73 ± 0.01 | 300 ± 26 | 458 ± 33 |
|  | 2.0×AA | 38 | 12/12 | 103.5 ± 1.2 | 0.79 ± 0.01 | 150 ± 19 | 167 ± 22 |

(continue)

| Clone | O_3_ | Leaf age | Leaf | *t*_leaf_ | *D*_leaf_ | *SD*_upp_ | *SD*_low_ |
| --- | --- | --- | --- | --- | --- | --- | --- |
|  |  |  | position | (μm) | (g cm^-3^) | (mm^-2^) | (mm^-2^) |
| Oxford | AA | 22 | 4/33 | 126.3 ± 1.7 | 0.59 ± 0.01 | 58 ± 23 | 333 ± 40 |
|  | AA | 27 | 6/32 | 135.7 ± 1.1 | 0.59 ± 0.00 | 75 ± 18 | 225 ± 54 |
|  | AA | 37 | 11/33 | 116.6 ± 0.4 | 0.67 ± 0.00 | 58 ± 15 | 250 ± 53 |
|  | AA | 53 | 15/32 | 126.6 ± 1.4 | 0.60 ± 0.01 | 58 ± 19 | 308 ± 42 |
|  | AA | 72 | 21/33 | 123.9 ± 1.5 | 0.68 ± 0.01 | 67 ± 14 | 250 ± 36 |
|  | AA | 90 | 29/33 | 126.7 ± 0.5 | 0.72 ± 0.00 | 33 ± 14 | 208 ± 38 |
|  | 1.5×AA | 23 | 3/30 | 120.0 ± 1.4 | 0.59 ± 0.01 | 100 ± 17 | 317 ± 34 |
|  | 1.5×AA | 27 | 5/24 | 136.7 ± 2.9 | 0.63 ± 0.01 | 50 ± 15 | 267 ± 47 |
|  | 1.5×AA | 40 | 6/26 | 113.2 ± 1.3 | 0.70 ± 0.01 | 92 ± 19 | 292 ± 50 |
|  | 1.5×AA | 58 | 13/24 | 112.9 ± 3.7 | 0.78 ± 0.02 | 100 ± 17 | 267 ± 51 |
|  | 1.5×AA | 71 | 20/30 | 114.9 ± 0.9 | 0.65 ± 0.01 | 75 ± 18 | 225 ± 41 |
|  | 1.5×AA | 92 | 22/26 | 118.9 ± 1.4 | 0.80 ± 0.01 | 117 ± 27 | 217 ± 27 |
|  | 2.0×AA | 21 | 3/19 | 122.8 ± 1.0 | 0.61 ± 0.01 | 75 ± 25 | 250 ± 38 |
|  | 2.0×AA | 27 | 4/25 | 111.7 ± 1.0 | 0.71 ± 0.01 | 108 ± 19 | 400 ± 54 |
|  | 2.0×AA | 34 | 7/17 | 113.8 ± 1.6 | 0.69 ± 0.01 | 117 ± 27 | 283 ± 24 |
|  | 2.0×AA | 51 | 15/19 | 133.0 ± 1.3 | 0.63 ± 0.01 | 58 ± 19 | 358 ± 42 |
|  | 2.0×AA | 63 | 18/25 | 136.1 ± 2.5 | 0.67 ± 0.01 | 67 ± 19 | 267 ± 33 |
|  | 2.0×AA | 68 | 16/17 | 123.4 ± 0.6 | 0.68 ± 0.00 | 50 ± 15 | 158 ± 26 |

**Table S4.** Average (±S.E.) mesophyll thickness (*t*_mes_, μm), fraction of intercellular air spaces (*F*_ias_, m^3^ m^-3^), mesophyll surface area exposed to intercellular air space per unit leaf area (*S*_mes_/*S*, m^2^ m^-2^), chloroplast surface area exposed to intercellular air space per unit leaf area (*S*_c_/*S*, m^2^ m^-2^), proportion of exposed mesophyll cell walls covered by chloroplasts (*S*_c_/*S*_mes_), cell wall thickness (*T*_cw_, μm), and chloroplast length (*L*_chl_, μm) and width (*W*_chl_, μm) in I-214 and Oxford poplar clones grown at three O_3_ levels (AA, ambient O_3_ concentration, 1.5×AA, 2.0×AA).

| Clone | O_3_ | Leaf age | *T*_mes_ | *F*_ias_ | *S*_mes_/*S* | *S*_c_/*S* | *S*_c_/*S*_mes_ | *T*_cw_ | *L*_chl_ | *W*_chl_ |
| --- | --- | --- | --- | --- | --- | --- | --- | --- | --- | --- |
|  |  |  | (μm) | (m^3^ m^-3^) | (m^2^ m^-2^) | (m^2^ m^-2^) | (fraction) | (μm) | (μm) | (μm) |
| I-214 | AA | 19 | 115.3 ± 1.9 | 0.14 ± 0.02 | 25.1 ± 1.6 | 20.3 ± 1.7 | 0.81 ± 0.03 | 0.25 ± 0.01 | 4.3 ± 0.3 | 2.6 ± 0.2 |
|  | AA | 34 | 99.6 ± 1.0 | 0.12 ± 0.01 | 22.3 ± 0.6 | 18.6 ± 1.2 | 0.84 ± 0.06 | 0.24 ± 0.01 | 4.3 ± 0.3 | 2.3 ± 0.2 |
|  | AA | 40 | 109.0 ± 0.6 | 0.15 ± 0.01 | 25.5 ± 1.8 | 14.9 ± 0.9 | 0.60 ± 0.04 | 0.31 ± 0.01 | 4.6 ± 0.2 | 2.8 ± 0.3 |
|  | AA | 66 | 106.1 ± 1.2 | 0.09 ± 0.01 | 19.1 ± 1.3 | 14.1 ± 1.1 | 0.75 ± 0.05 | 0.36 ± 0.02 | 4.2 ± 0.2 | 2.6 ± 0.2 |
|  | AA | 87 | 106.6 ± 1.9 | 0.13 ± 0.01 | 23.3 ± 0.7 | 8.4 ± 1.2 | 0.35 ± 0.04 | 0.61 ± 0.05 | 4.7 ± 0.3 | 2.3 ± 0.1 |
|  | AA | 92 | 86.9 ± 1.7 | 0.12 ± 0.01 | 20.5 ± 1.5 | 7.5 ± 0.6 | 0.37 ± 0.02 | 0.60 ± 0.08 | 4.7 ± 0.6 | 2.0 ± 0.4 |
|  | AA | 101 | 85.1 ± 1.9 | 0.14 ± 0.02 | 19.5 ± 1.7 | 9.3 ± 0.5 | 0.48 ± 0.02 | 0.62 ± 0.09 | 4.3 ± 0.6 | 2.5 ± 0.7 |
|  | 1.5×AA | 21 | 96.8 ± 1.2 | 0.11 ± 0.01 | 19.4 ± 0.8 | 17.3 ± 1.3 | 0.89 ± 0.04 | 0.24 ± 0.02 | 4.8 ± 0.2 | 3.1 ± 0.1 |
|  | 1.5×AA | 32 | 101.6 ± 0.9 | 0.10 ± 0.00 | 15.9 ± 0.7 | 14.0 ± 1.1 | 0.89 ± 0.09 | 0.22 ± 0.02 | 4.4 ± 0.2 | 2.8 ± 0.2 |
|  | 1.5×AA | 37 | 99.5 ± 0.6 | 0.13 ± 0.01 | 19.2 ± 1.0 | 18.9 ± 1.6 | 0.98 ± 0.05 | 0.29 ± 0.02 | 4.6 ± 0.2 | 2.1 ± 0.1 |
|  | 1.5×AA | 59 | 97.3 ± 1.7 | 0.18 ± 0.01 | 17.8 ± 1.2 | 10.2 ± 1.1 | 0.57 ± 0.04 | 0.46 ± 0.05 | 4.5 ± 0.2 | 2.2 ± 0.1 |
|  | 1.5×AA | 65 | 102.3 ± 0.5 | 0.11 ± 0.01 | 14.5 ± 1.5 | 10.0 ± 1.4 | 0.70 ± 0.08 | 0.38 ± 0.02 | 4.9 ± 0.8 | 2.2 ± 0.1 |
|  | 1.5×AA | 84 | 103.1 ± 1.6 | 0.18 ± 0.01 | 19.1 ± 0.8 | 5.7 ± 0.4 | 0.30 ± 0.02 | 0.50 ± 0.02 | 4.4 ± 0.4 | 2.6 ± 0.1 |
|  | 1.5×AA | 89 | 127.8 ± 2.4 | 0.32 ± 0.01 | 25.5 ± 0.9 | 4.1 ± 0.5 | 0.16 ± 0.02 | 0.55 ± 0.06 | 4.0 ± 0.4 | 2.7 ± 0.2 |
|  | 2.0×AA | 17 | 110.9 ± 1.3 | 0.15 ± 0.02 | 25.7 ± 2.3 | 16.9 ± 1.1 | 0.68 ± 0.06 | 0.44 ± 0.04 | 2.9 ± 0.2 | 2.1 ± 0.2 |
|  | 2.0×AA | 23 | 83.1 ± 0.6 | 0.15 ± 0.01 | 15.7 ± 1.6 | 6.6 ± 0.3 | 0.44 ± 0.04 | 0.46 ± 0.03 | 2.9 ± 0.1 | 1.8 ± 0.1 |
|  | 2.0×AA | 26 | 88.3 ± 0.7 | 0.25 ± 0.02 | 23.5 ± 0.6 | 10.0 ± 1.0 | 0.38 ± 0.04 | 0.44 ± 0.03 | 2.9 ± 0.2 | 1.9 ± 0.2 |
|  | 2.0×AA | 26 | 121.8 ± 1.1 | 0.23 ± 0.03 | 26.7 ± 1.8 | 9.3 ± 0.3 | 0.40 ± 0.01 | 0.42 ± 0.03 | 3.6 ± 0.3 | 2.1 ± 0.3 |
|  | 2.0×AA | 32 | 85.4 ± 1.0 | 0.18 ± 0.01 | 20.0 ± 1.0 | 11.7 ± 0.8 | 0.59 ± 0.05 | 0.45 ± 0.04 | 4.4 ± 0.4 | 1.9 ± 0.2 |
|  | 2.0×AA | 34 | 93.4 ± 1.2 | 0.18 ± 0.04 | 20.4 ± 2.7 | 5.6 ± 0.4 | 0.29 ± 0.02 | 0.51 ± 0.05 | 3.8 ± 0.5 | 2.4 ± 0.5 |
|  | 2.0×AA | 38 | 87.2 ± 0.6 | 0.33 ± 0.02 | 30.0 ± 0.8 | 2.6 ± 0.1 | 0.08 ± 0.02 | 0.60 ± 0.03 | 4.5 ± 0.1 | 2.2 ± 0.2 |

(continue)

| Clone | O_3_ | Leaf age | *T*_mes_ | *F*_ias_ | *S*_mes_/*S* | *S*_c_/*S* | *S*_c_/*S*_mes_ | *T*_cw_ | *L*_chl_ | *W*_chl_ |
| --- | --- | --- | --- | --- | --- | --- | --- | --- | --- | --- |
|  |  |  | (μm) | (m^3^ m^-3^) | (m^2^ m^-2^) | (m^2^ m^-2^) | (fraction) | (μm) | (μm) | (μm) |
| Oxford | AA | 22 | 111.8 ± 1.3 | 0.31 ± 0.03 | 24.8 ± 1.3 | 16.9 ± 1.4 | 0.68 ± 0.03 | 0.23 ± 0.01 | 5.1 ± 0.4 | 1.9 ± 0.1 |
|  | AA | 27 | 120.4 ± 1.7 | 0.34 ± 0.02 | 27.6 ± 1.3 | 20.1 ± 1.2 | 0.74 ± 0.05 | 0.22 ± 0.01 | 4.9 ± 0.3 | 1.7 ± 0.2 |
|  | AA | 37 | 101.3 ± 0.6 | 0.33 ± 0.02 | 23.3 ± 0.7 | 11.7 ± 1.2 | 0.50 ± 0.05 | 0.44 ± 0.02 | 4.8 ± 0.2 | 1.4 ± 0.1 |
|  | AA | 53 | 111.0 ± 1.4 | 0.35 ± 0.01 | 27.2 ± 2.3 | 15.8 ± 2.3 | 0.60 ± 0.09 | 0.42 ± 0.02 | 4.4 ± 0.2 | 1.6 ± 0.1 |
|  | AA | 72 | 110.4 ± 1.3 | 0.39 ± 0.02 | 28.9 ± 2.5 | 12.8 ± 1.2 | 0.46 ± 0.06 | 0.38 ± 0.02 | 4.7 ± 0.2 | 1.1 ± 0.2 |
|  | AA | 90 | 111.9 ± 1.0 | 0.24 ± 0.01 | 27.3 ± 1.3 | 7.0 ± 0.9 | 0.26 ± 0.04 | 0.41 ± 0.02 | 4.9 ± 0.3 | 1.4 ± 0.1 |
|  | 1.5×AA | 23 | 104.4 ± 1.4 | 0.29 ± 0.01 | 16.7 ± 1.1 | 12.1 ± 1.6 | 0.75 ± 0.11 | 0.33 ± 0.02 | 4.6 ± 0.1 | 1.6 ± 0.1 |
|  | 1.5×AA | 27 | 120.4 ± 2.8 | 0.38 ± 0.02 | 23.5 ± 0.3 | 21.5 ± 0.9 | 0.91 ± 0.03 | 0.36 ± 0.03 | 4.4 ± 0.2 | 1.7 ± 0.1 |
|  | 1.5×AA | 40 | 100.3 ± 1.3 | 0.32 ± 0.02 | 21.0 ± 2.3 | 13.4 ± 0.9 | 0.67 ± 0.06 | 0.38 ± 0.02 | 3.5 ± 0.2 | 1.5 ± 0.1 |
|  | 1.5×AA | 58 | 96.9 ± 3.8 | 0.27 ± 0.03 | 15.3 ± 1.2 | 7.3 ± 0.3 | 0.49 ± 0.03 | 0.37 ± 0.02 | 3.9 ± 0.2 | 1.6 ± 0.1 |
|  | 1.5×AA | 71 | 97.2 ± 0.8 | 0.19 ± 0.01 | 20.1 ± 2.5 | 9.6 ± 1.2 | 0.50 ± 0.05 | 0.32 ± 0.03 | 3.2 ± 0.5 | 1.6 ± 0.1 |
|  | 1.5×AA | 92 | 104.4 ± 0.9 | 0.33 ± 0.01 | 25.6 ± 0.6 | 10.2 ± 0.5 | 0.40 ± 0.03 | 0.37 ± 0.03 | 2.2 ± 0.2 | 1.8 ± 0.3 |
|  | 2.0×AA | 21 | 109.0 ± 0.7 | 0.33 ± 0.01 | 27.4 ± 1.8 | 11.9 ± 0.6 | 0.44 ± 0.03 | 0.35 ± 0.03 | 3.5 ± 0.2 | 2.0 ± 0.3 |
|  | 2.0×AA | 27 | 97.7 ± 1.0 | 0.34 ± 0.02 | 26.8 ± 1.9 | 10.1 ± 0.3 | 0.39 ± 0.03 | 0.34 ± 0.03 | 4.1 ± 0.1 | 1.6 ± 0.2 |
|  | 2.0×AA | 34 | 101.9 ± 1.2 | 0.22 ± 0.01 | 15.7 ± 1.5 | 8.3 ± 1.0 | 0.55 ± 0.06 | 0.54 ± 0.04 | 3.2 ± 0.1 | 1.3 ± 0.1 |
|  | 2.0×AA | 51 | 119.0 ± 1.0 | 0.42 ± 0.01 | 20.6 ± 0.9 | 5.0 ± 0.6 | 0.25 ± 0.03 | 0.52 ± 0.03 | 3.0 ± 0.2 | 1.4 ± 0.1 |
|  | 2.0×AA | 63 | 119.7 ± 2.6 | 0.35 ± 0.01 | 30.1 ± 1.6 | 7.1 ± 0.6 | 0.24 ± 0.03 | 0.40 ± 0.03 | 2.5 ± 0.2 | 1.7 ± 0.2 |
|  | 2.0×AA | 68 | 110.6 ± 0.7 | 0.37 ± 0.00 | 17.5 ± 0.8 | 5.2 ± 0.4 | 0.30 ± 0.02 | 0.47 ± 0.03 | 3.0 ± 0.4 | 1.8 ± 0.2 |

**Supplementary references**

Genty B, Briantais J-M, Baker NR. 1989. The relationship between the quantum yield of photosynthetic electron transport and quenching of chlorophyll fluorescence. *Biochimica et Biophysica Acta (BBA) - General Subjects* **990**: 87–92.

Gilbert ME, Pou A, Zwieniecki MA, Holbrook NM. 2012. On measuring the response of mesophyll conductance to carbon dioxide with the variable J method, *Journal of Experimental Botany* **63**: 413–425.

Laisk A, Loreto F. 1996. Determining photosynthetic parameters from leaf CO_2_ exchange and chlorophyll fluorescence - ribulose-1,5-bisphosphate carboxylase oxygenase specificity factor, dark respiration in the light, excitation distribution between photosystems, alternative electron transport rate, and mesophyll diffusion resistance. *Plant Physiology* **110**: 903–912.

Sharkey TD. 1988. Estimating the rate of photorespiration in leaves. *Physiologia Plantarum* **73**: 147–152.
